# Simulation of Force Spectroscopy Experiments on Galacturonic Acid Oligomers

**DOI:** 10.1371/journal.pone.0107896

**Published:** 2014-09-17

**Authors:** Justyna Cybulska, Agnieszka Brzyska, Artur Zdunek, Krzysztof Woliński

**Affiliations:** 1 Institute of Agrophysics, Polish Academy of Sciences, Lublin, Poland; 2 Jerzy Haber Institute of Catalysis and Surface Chemistry, Polish Academy of Sciences, Kraków, Poland; 3 Department of Chemistry, Maria Curie-Skłodowska University, Lublin, Poland; University of Calgary, Canada

## Abstract

Pectins, forming a matrix for cellulose and hemicellulose, determine the mechanics of plant cell walls. They undergo salient structural changes during their development. In the presence of divalent cations, usually calcium, pectins can form gel-like structures. Because of their importance they have been the subject of many force spectroscopy experiments, which have examined the conformational changes and molecular tensions due to external forces. The most abundant unit present in the pectin backbone is polygalacturonic acid. Unfortunately, experimental force spectroscopy on polygalacturonic acid molecules is still not a trivial task. The mechanism of the single-molecule response to external forces can be inferred by theoretical methods. Therefore, in this work we simulated such force spectroscopy experiments using the Enforced Geometry Optimization (EGO) method. We examined the oligomeric (up to hexamer) structures of α-D-galacturonic acid exposed to external stretching forces. The EGO simulation of the force spectroscopy appropriately reproduced the experimental course of the enforced conformational transition: chair →inverted chair via the twisted boat conformation(s) in the pyranose ring of α-D-galacturonic acid. Additionally, our theoretical approach also allowed to determine the minimum oligomer size adequate for the description of nano-mechanical properties of (poly)-α-D-galacturonic acid.

## Introduction

Recently, atomic force microscopy (AFM) has proven itself as a very valuable tool for the investigation of the nano-structure and nano-mechanical properties of single biomolecules or assemblies, including cell walls. The AFM technique can be useful for analyzing the effect of different modifying agents on polysaccharide structures [1], [2], cellulose in cell wall model materials [3] and cellulose in the cell wall of apples in relation to their texture [4]. It has also been shown that the chemical composition of model cell walls has a significant influence on their surface topographic parameters (RMS roughness, height, ironed surface) [3], [5].

Pectins are particularly interesting molecular systems because they constitute a matrix for cellulose and hemicellulose microfibrils. These biomolecules undergo salient structural changes during their development. In the presence of divalent cations, usually calcium, pectins can form gel-like structures. Polygalacturonic acid - the main unit of the pectin backbone - is particularly useful in food production, chemical, cosmetic and pharmaceutical industries. Due to the presence of reactive carboxyl groups, (poly)galacturonic acid molecules may undergo several chemical reactions, modifications and naturally-driven enzymatic reactions. Enzymatic activeness results primarily in the depolymerization of polygalacturonic acid and its de-esterification [6]. These processes have a significant influence on rheological properties, swelling abilities, gelling under different pH and temperature conditions and networking properties, and consequently on the mechanical properties of pectin compounds.

One of the first AFM experimental analyses of pectin nano-structures was conducted by Yang et al. [7], who showed the fraction of water soluble pectin extracted from peach parenchyma. On the basis of geometrical dimensions of pectin chains it was shown that parallel linkages or intertwists between the basic units of the pectin molecules occurred [7]. Kirby, McDougall and Morris [8] presented AFM images of pectin extracted from tomato and sugar beet and an analysis of pectin chain aggregation levels. The analysis of pectin chain length allowed for the determination of calcium concentration, which hinders the depolymerization process [9]. Yang, Chen, An and Lai [10] observed that the differences in suppleness between three varieties of peaches may be due to the different lengths of pectin chains. The first attempts to investigate the mechanical properties of pectin with force spectroscopy showed interactions between ramnogalacturonan chains and galactose. However, due to the small size (up to a few dozen nanometers in length), the possibilities for testing their mechanical properties are very limited [11]. One solution to this problem is to use molecular modeling to define the resistance of polygalacturonic acid particles for external interactions [12], [13].

The stretching of the individual molecules of cyclic compounds, polysaccharides, proteins and nucleic acids can be simulated in order to investigate the relationship between the applied forces and their molecular deformation [14], [15], [16]. Molecular modeling allows for the prediction of molecule transformations into different isomeric forms. For the obtained isomers, it is also possible to generate a spectrum (e.g., IR, Raman or NMR) and experimentally verify the result. Woliński and Baker [17] proposed a theoretical model of Enforced Geometry Optimization (EGO) simulating the effects of external forces acting on molecules, which also takes place in AFM force spectroscopy. The EGO model was used to examine enforced conformation changes in the pyranose ring observed in several AFM experiments on individual polysaccharide molecules [16]. The enforced geometry optimization of the stretched pyranose ring resulted in a change of the molecule conformation from a chair into a boat. This change is caused by the force transmission of a torque on glycosidic bonds, which work as mechanical levers and generate a rotational moment under the influence of external forces. Additionally, the EGO model was efficiently applied for generating new, previously unknown isomers of azobenzene (C_12_N_2_H_10_) and stilbene (C_14_H_12_) [17], [18], and also for the investigation of forced disintegration and recombination of a series of six-elemental rings: 1,3,5-trioxane, 1,3,5-triazine, cyclohexane and benzene [16].

To the best of our knowledge, the theoretical model of polymers with a backbone composed of polygalacturonic acid, has not yet been studied with the aim of revealing their mechanical properties. This issue is important for two reasons. Firstly, a theoretical model will allow for the prediction of conformation change(s) in the (poly)galacturonic acid chain. Secondly, the model will help in future interpretations of AFM force spectroscopy experiments on pectic molecular systems. To summarize, the aim of this study was to model future AFM experiments involving stretching polygalacturonic acid molecules in order to define some of their mechanical properties.

## Methods

### Theoretical considerations

The structure of pectin compounds includes fragments mostly composed of non-branched chains of homogalacturonans. From a chemical perspective, homogalacturonans are made during the polymerization of α-D-galacturonic acid with occasional rhamnosyl residues that put a kink in the chain [19]. In homogalacturonan c.a. 100 monomers of galacturonic acid make up the ‘smooth region’ of pectin [20].

The preferred conformation of α-D-galacturonic acid in homogalacturonan is the chair conformation ^4^C_1_. The most thermodynamically stable form of carbohydrates are with D-pyranose rings. Carbon atoms in rings tend to form bonds at an angle of c.a. 109°. With external forces the conformation may be transformed into a boat form, where carbon atoms (C1 and C4) lie above the ring surface. The form is not stable due to its angular and torsion strains. Other units of α-D-polygalacturonic acid are bound with a glycosidic bond by atoms C1–O1–C4’.

**Figure 1:**
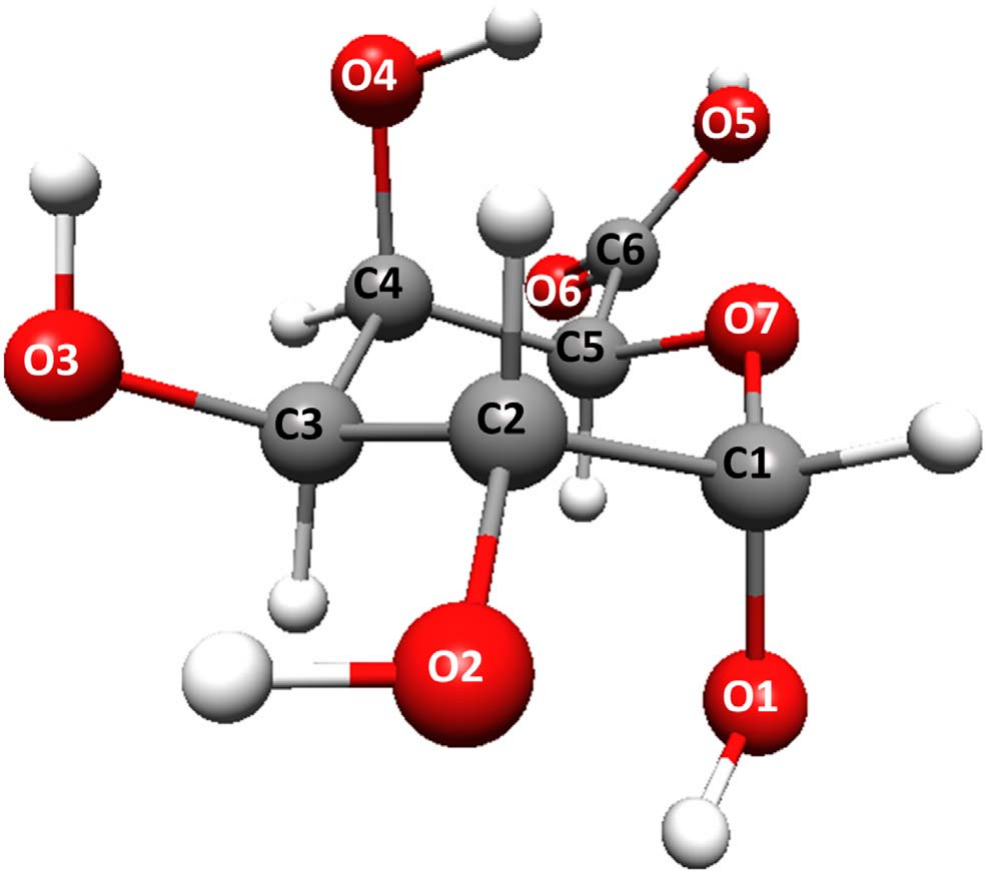
The atom numbering in α-D-galacturonic acid monomer.

The main structural unit of pectin, α-D-galacturonic acid, was the object of theoretical considerations in this study (see Figure 1). A large number of factors, such as the degree and pattern of esterification, charge density and properties of solvent can affect the structural organization of (poly)galacturonic acid in a solution. The geometries of individual monosaccharide units are basically rigid. However, there are several effects (e.g. steric hindrance restricting the rotation around chemical bonds, electrostatic effect and hydrogen bonding) which can additionally have effects on the conformation and structure of a single polysaccharide chain [21]. All these factors are difficult to analyze together even in molecular modeling studies. The correct generation of the starting geometries determines the quality of further calculations. Therefore, in our study the initial galacturonic acid structures, based on X-ray experimental data [22], were built using PQSMol software. The internal H-bond orientations in the oligosaccharide chain arise from its helix structure. The initial geometries were optimized at a DFT/B3LYP level with the 6-311G-dp basis set.

All calculations presented in this work were performed using the PQS package (Parallel Quantum Solutions, USA). We used the DFT approach (Density Functional Theory) due to its accurate prediction of molecular geometries [23]. The popular (preferred) hybrid B3LYP functional with the widely used standard 6-311G(d,p) basis set were applied. This basis set provides accurate geometry and electronic properties for a wide range of organic compounds [24].

In the present work the standard geometry optimization was performed for monomers and the subsequent oligomers up to the hexamer of α-D-galacturonic acid. Vibrational analysis confirmed that in all cases, stable structures (local minimum at PES) were obtained.

Geometry optimization in the presence of external forces (EGO) was performed for α-D-galacturonic acid oligomers from monomer up to hexamer. In these EGO calculations (simulating AFM experiments) the input (starting) structures were those obtained from the standard geometry optimization as described above. In all cases, external forces were applied to the terminal oxygen atoms in positions C1 and C4 of the chain/molecule (potential location of glycosidic bond with another saccharide unit in an oligomer molecule). This configuration simulates forces acting on an individual α-D-galacturonic acid unit in a polymer chain during stretching. For the monomer, dimer and trimer these forces were in the range of *f* = 0.02–0.08 au (1 au = 82.39 p nN) with a step of 0.005 au (0.41 nN). This allowed us to determine the minimum forces that caused individual conformational transitions. For higher oligomers (tetramer, pentamer and hexamer) EGO calculations were performed for only one magnitude of the external force of 0.07 au (5.77 nN). Such a large force induced all possible transitions in the galacturonic acid oligomers considered here.

The geometry optimization procedure in the presence of external forces ends after having met standard compliance criteria on the gradient, geometry displacement and energy change values, predefined in the PQS optimization package. The new, stressed structures were relaxed, i.e. re-optimized after removing external forces. The stability of the new, relaxed structures was verified by vibrational analysis. It should be pointed out that the EGO method allows prediction of the structures that may result from the AFM experiment. In the stretched (final) structures, the deformation of pyranose ring/conformational transition(s) in saccharide oligomers are caused by external forces (and these structures are allowed to occur in AFM stretching experiments). The relaxation process is mainly to check whether or not these structural changes are permanent.

## Results and Discussion

Marszałek et al. [25] proved experimentally that the forced (due to stretching) conformational transitions in polysaccharides depend on the nature of the glycosidic linkages present in the carbohydrate chain and their spatial alignment. Polymers that possess only equatorial glycosidic linkages (such as cellulose) do not show any conformational changes as a result of stretching. Generally, force-induced conformational transitions are observed in polysaccharides with axially-linked monomer units. The number of these structural changes is related to the sum of glycosidic and aglycone bonds in the axial position (per residue).

The most important structural feature of the poly-α-(1–4)galacturonic acid is the axial orientation of both the glycosidic (C1-O1) and aglycone (O1-C4) bonds. These bonds act as mechanical levers generating the torque of the ring which enables two force-driven conformational transitions [26].

One aim of the present work was to determine the mechanical properties of the α-D-galacturonic acid. Since experimental force spectroscopy on galacturonic acid molecules is still not a trivial task, and theoretical simulations of structural changes in α-D-galacturonic acid oligomers (up to hexamer) were completed using the EGO model.

The optimized α-D-galacturonic acid oligomer structures (with ^4^C_1_ conformation of each saccharide unit) were subjected to stretching by external forces. The forces were always applied to oxygen atoms in positions O1 and O4 in the terminal (axially arranged) –OH groups which had the potential to form glycosidic bonds. The applied forces had opposite directions and worked along a straight line connecting the two selected oxygen atoms.

### Monomer, dimer and trimer structures under external stretching forces

**Figure 2:**
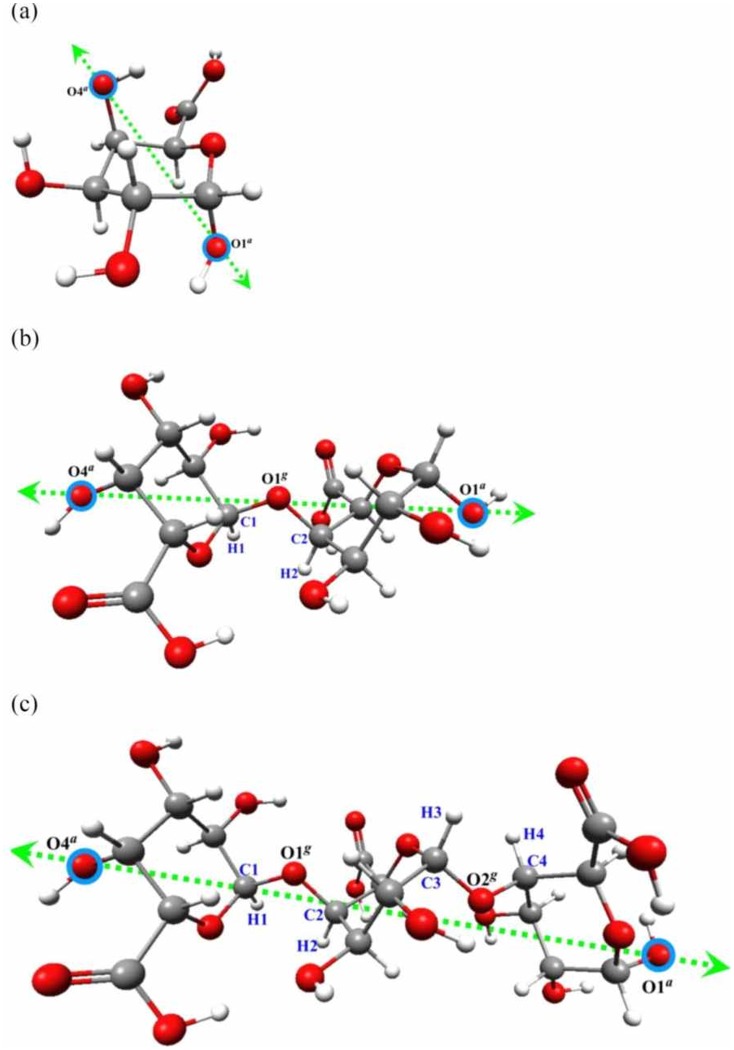
The scheme of stretching forces. The scheme of external stretching force actions on O1*^a^* and O4*^a^* oxygen atoms in α-D-galacturonic acid (a) monomer, (b) dimer, (c) trimer. O*^g^* indicates the oxygen atom involved in the glycosidic bond. The characteristic dihedral angles are defined as: φ1 = H1C1O1*^g^*C2, ψ1 = C1O1*^g^*C2H2 and φ2 = H3C3O2*^g^*C4, ψ2 = C3O2*^g^*C4H4).

The initial monomer, dimer and trimer structures and the mode of external force actions are presented in Figure 2. The structures obtained in the presence of external forces were subjected to the relaxation process, i.e. standard geometry optimization without any external forces. To confirm that the obtained structures (after relaxation) were stable, a vibrational analysis was performed. Detailed numerical data for monomer, dimer and trimer structures are found in Tables S1–S3.

**Figure 3:**
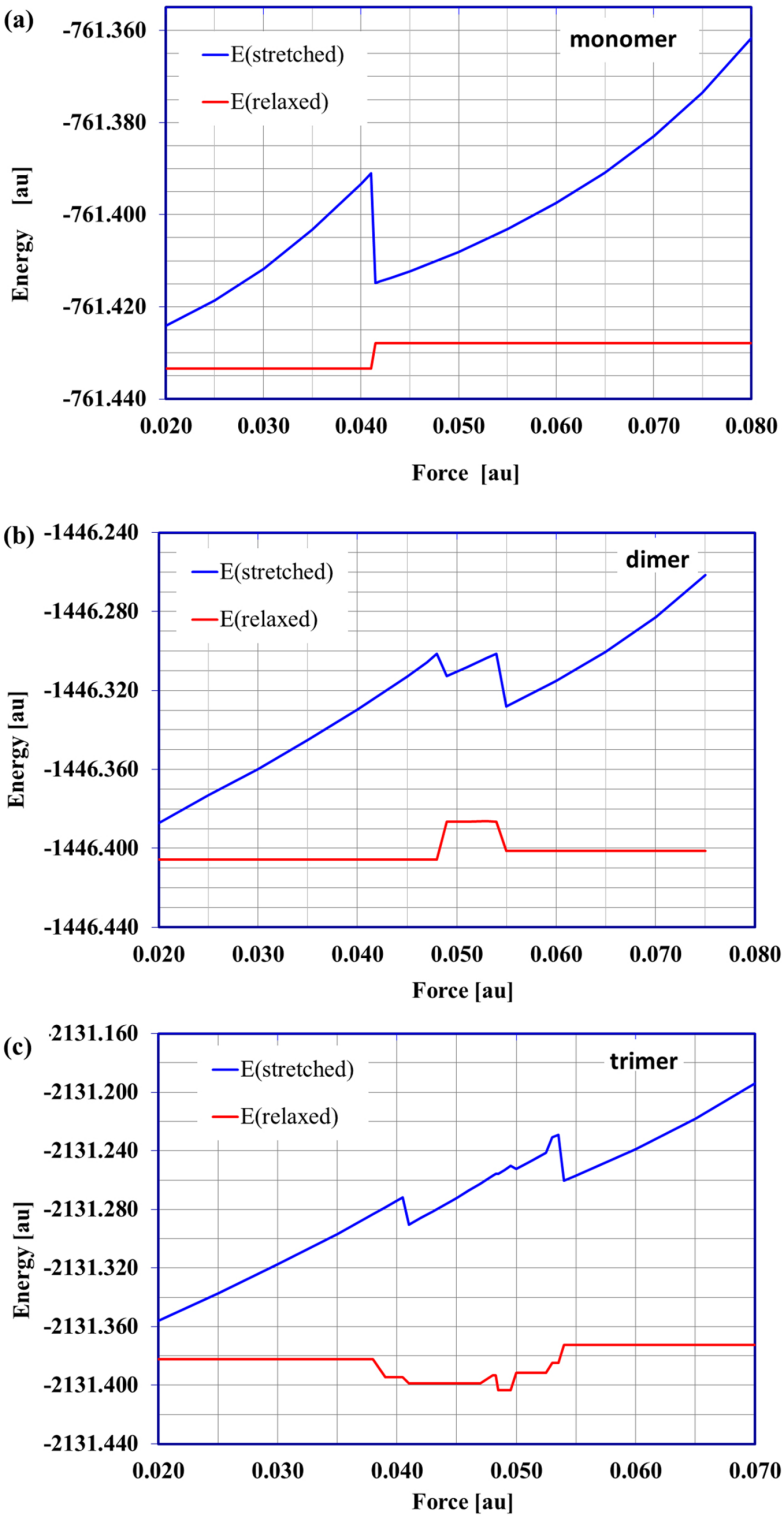
The energy of stretched and relaxed molecules. The energy of the stretched (forced) and corresponding relaxed (a) monomer, (b) dimer, (c) trimer structures as a function of applied external force. 1au = 82.39 nN.

**Figure 4:**
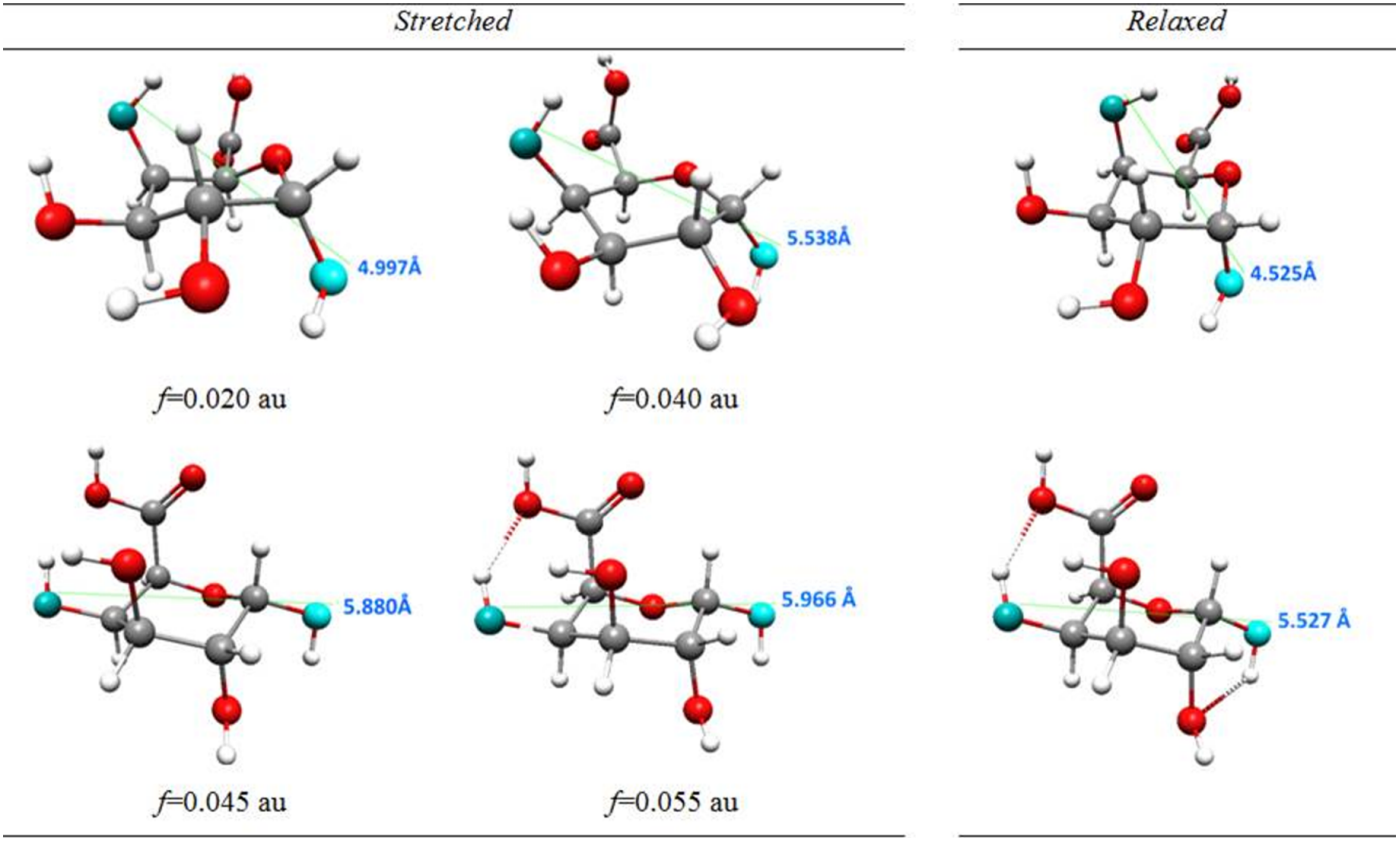
Monomer structures obtained as a result of external forces. The selected structures obtained as a result of external forces applied to oxygen atoms in 1,4 position in ^4^C_1_ conformer of α-D-galacturonic acid molecule with a marked distance between oxygen atoms O1 and O4.

In the case of the α-D-galacturonic acid monomer the minimal external stretching force needed to induce the permanent conformational changes in the molecule is 0.041 au (3.38 nN). The lower external forces caused only (temporary) deformations and when the external force was removed the molecule returned to its stable starting structure (with ^4^C_1_ conformation). Applying a stretching force of at least 0.041 au induced the direct conformational transition *chair ^4^C_1_ →inverted chair ^1^C_4_*. Such a structural change should entail a significant molecular energy shift and it did, as shown in the relationships between the energy of the stressed (stretched) and corresponding *relaxed* structures and applied stretching force (Figure 3a). This plot clearly shows only one possible conformational transition. Note that for *f≥*0.041 au the O4*^a^*O1*^a^* distance (Table S1) of the relaxed structure is equal to about 5.5 Å and corresponds to the inverted chair isomer. Forces >0.08 au (6.59 nN) broke the bonds C1O1 and C4O4 in the monomer molecule. The selected stressed and relaxed monomer structures are shown in Figure 4.

**Figure 5:**
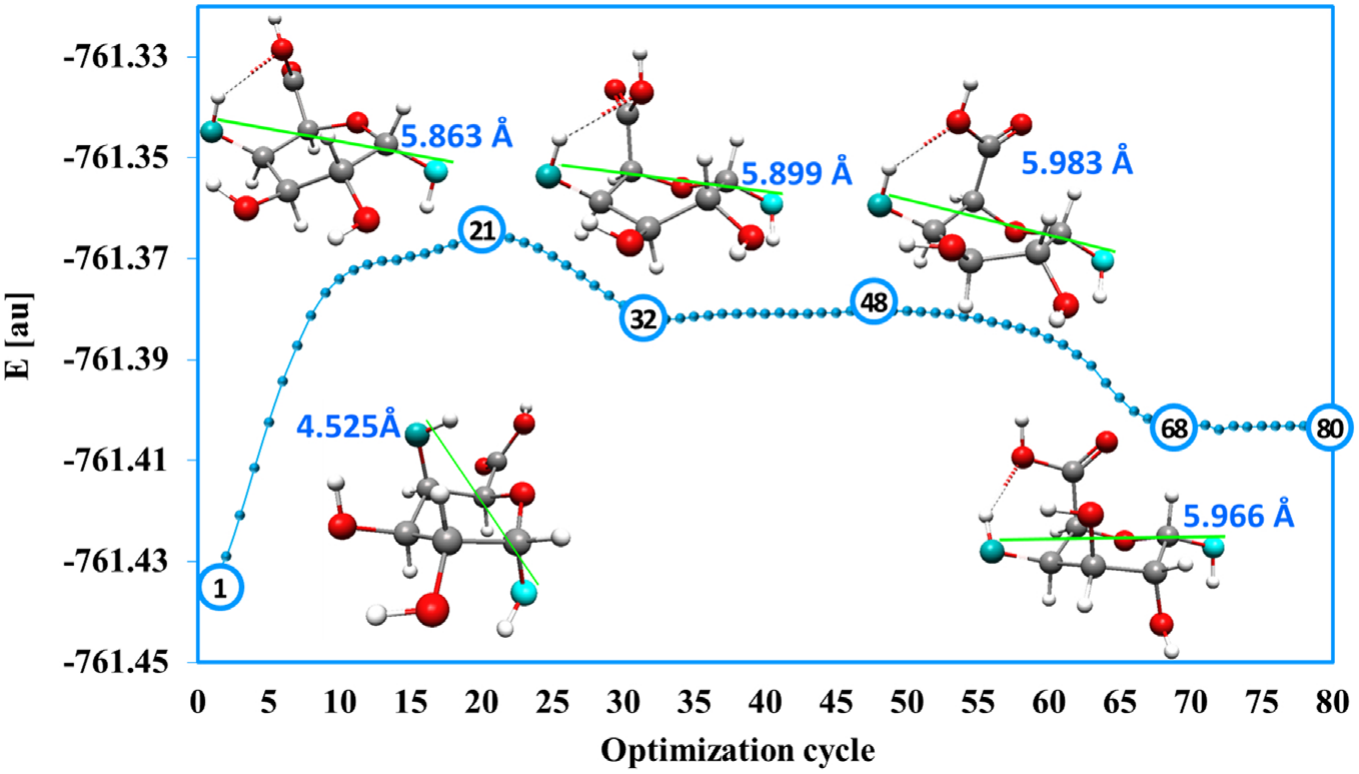
History of ^4^C_1_ conformer optimization. History of ^4^C_1_ conformer optimization in the presence of external force *f = 0.055 au* (*f = 4.53 nN*) applied to atoms O1 and O4 with the structures from selected optimization cycles (1, 21, 32, 48, 68).

The standard geometry optimization pathway (energy changes with optimization cycles) does not represent any real reaction path. However, in the presence of external forces the geometry optimization history plot delivers some interesting information about the reaction mechanism. History of the enforced geometry optimization for the monomer with the stretching external force of 0.055 au (4.53 nN) showed a number of characteristic points (Figure 5). They corresponded to the maximum and minimum of the molecular energy (at optimization cycles 21, 32, 48 and 68). The energy of the system increased from the starting value at cycle 1, reaching a first maximum at cycle 21 and then decreasing to a minimum at cycle 32. This can be viewed as crossing the first energy barrier i.e. passing the first transition state. The energy slightly increased from cycle 32, reaching a second maximum at cycle 48 and then decreased to a second minimum at cycle 68. Again, this can be related to passing through the second transition state. Note that the first energy barrier was much higher than the second. This means that if an applied external force (here 0.055 au) was big enough to move a molecular system over the first barrier, then the second (lower) barrier would also be overcome automatically. These considerations were confirmed in the relaxation process where the lowest energy structures (cycle 32 and 68) were re-optimized after removing external forces and the highest energy structures (cycles 21 and 48) were taken as the initial guess for the transition state searching procedure. Relaxing structures ‘32’ and ‘68’ led to the stable twisted boat and inverted stable chair conformers, respectively. From the maximum energy structures ‘21’ and ‘48’, two transition states (each connecting two corresponding local minimum) were verified by vibrational analysis. It should be emphasized, however, that very similar plots were obtained for all external forces in the range of 0.041–0.08 au (i.e. from the minimum force that causes an inversion chair ^4^C_1_ → chair ^1^C_4_ to the minimum force that breaks the C1O1 and C4O4 bonds). They all had two local minima and two local maxima. Each external force of a given magnitude from this range induced the *direct* ^4^C_1_ → ^1^C_4_ inversion, the mechanism which involves an intermediate twisted boat structure. Energy values for molecular structures obtained in the presence of external forces and corresponding relaxed structures are presented in Table 1.

**Table 1:** The values of energy and O4*^a^*O1*^a^* distance corresponding to molecular structures obtained in selected cycles of forced geometry optimization and the energy of *relaxed* structures with the lowest harmonic vibration frequencies.

No. Cycle	Stretched structure		*Relaxed* structure		
	*E*	O4*^a^*O1*^a^*	*E*	*ν_min_*	O4*^a^*O1*^a^*
	[au]	[Å]	[au]	[cm^−1^]	[Å]
1	–761.43335	4.524	–761.43335	37.689	4.524
21	–761.36559	5.863	–761.41686	–67.98 (TS)	4.949
32	–761.38167	5.899	–761.42078	39.53	4.296
48	–761.38037	5.987	–761.41468	–69.96 (TS)	5.116
68	–761.40316	5.966	–761.42791	47.79	5.527

Experimental works [26] concerning conformation changes in individual α -D-galacturonic acid molecules imposed by external forces showed that the transformation ^4^C_1_ → ^1^C_4_ takes place through the twisted boat conformation, as a *two-step* process. First, a smaller force transforms the ^4^C_1_ chair conformer into a twisted boat and then a greater force is needed to make the final transition into the ^1^C_4_ chair. In contrast, according to EGO calculations for the monomer of galacturonic acid presented here, the ^4^C_1_ → ^1^C_4_ chair inversion should be a *one-step* process because it was directly induced by one force (with a magnitude of at least of 0.041 au) clearly shown in Figure 3a. However, the EGO calculations showed that *the mechanism* of this direct inversion indeed involved an intermediate transition into a twisted boat structure, which cannot be seen on the energy-force plot (Figure 3a) because the first energy barrier separating ^4^C_1_ and twisted boat was higher than the second barrier between the twisted boat and ^1^C_4_. It should also be mentioned that the enforced chair inversion reaction simulated by the EGO, predicted the reorientation of the hydroxyl groups in positions 2 and 3 of the saccharide ring from equatorial to axial.

Analogous mechanisms of conformation changes resulting from molecule stretching are also predicted using molecule dynamics simulations with the Amber99 and Amber-Glycam0 force fields [27] as well as with DFT computations based on constrained geometry optimization (CGO) with a *frozen* distance between oxygen atoms in positions O1 and O4 conducted in the range of 4.45–6.0 Å [28].

Table 2 presents a comparison of the results obtained using different theoretical methods with AFM experimental data. The EGO model predicted essentially the same mechanism for the conformational transformation as the molecular dynamics and constrained geometry optimization calculations. However, the theoretical (EGO) lengths of chair (^1^C_4_) and twisted boat conformers of a (stretched) α-D-galacturonic acid monomer were somewhat overestimated compared to experimental results.

**Table 2:** Experimental (AFM experiment) and theoretical (MD method, EGO model) lengths of α-D-galacturonic acid conformers.

Method	L^4^C_1_ [Å]	L twisted boat [Å]	L^1^C_4_ [Å]
AFM(1)	4.500	4.901	5.405
MD(2)	4.7	5.2–5.5	5.7
DFT/6–311++G-dp(3)	4.592	5.176	5.547
EGO	4.525	5.983(4)	5.966
	4.525	5.116(5) ^,^ (4)	5.527(5)

(1) experimental data [26].

(2) data obtained with molecular dynamics methods [27].

(3) data obtained in the process of geometry optimization with the frozen distance between glycosidic oxygen atoms [28].

(4) structure marked as 48 in the Figure 4.

(5) values for the relaxed structures.

For the dimer structure the situation became slightly more complicated. The stretched/relaxed energy profile (Figure 3b), displayed two distinctive conformational changes. This plot could be naturally divided into three regions. In the first region (0.02≤*f*≤0.048 au; 1.65≤*f*≤3.95 nN) there were no permanent conformational changes. The relaxation process led to a starting dimer structure with chair conformations of both saccharide units (the characteristic O4*^a^*O1*^g^* and O1*^g^*O1*^a^* distances equal about 4.5 Å) (Table S2).

**Figure 6:**
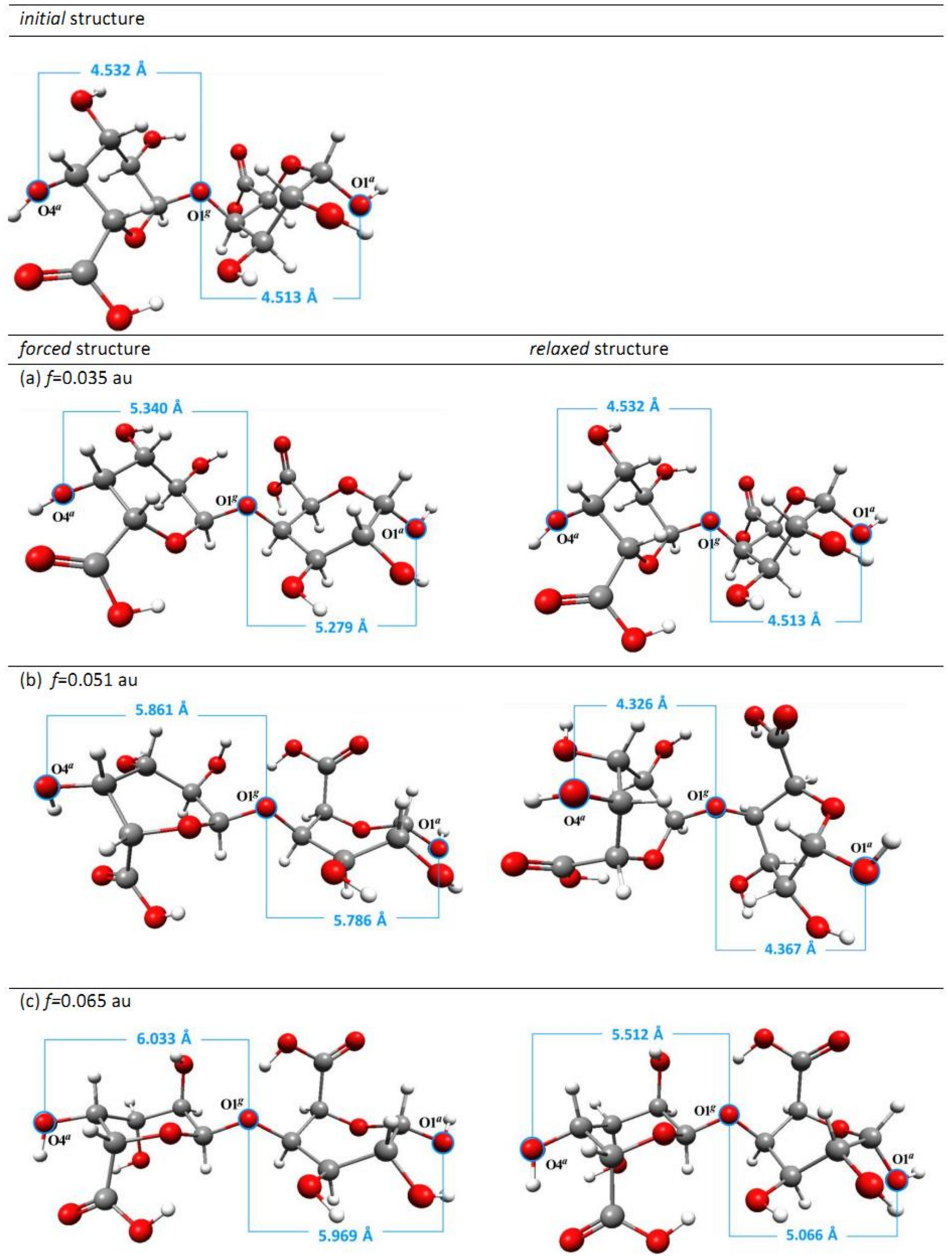
Stretched and relaxed dimer structures. The selected stretched and corresponding relaxed dimer structures with marked oligomer unit lengths.

The second region corresponds to the external forces in the range of 0.049–0.054 au (4.04–4.45 nN). The relaxation of the stretched dimer molecules obtained with these force magnitudes yielded the twisted boat conformation of both galacturonic acid units. The O4*^a^*O1*^g^* and O1*^g^*O1*^a^* distances are about 4.3 Å and refer to the twisted boat conformation (called *type 1* here). In the last region revealed in Figure 3b for the stretching forces of 0.055–0.075 au (4.53–6.18 nN) there were two types of conformational transitions: namely, *chair →inverted chair* (for the relaxed structure O4*^a^*O1*^g^*≈5.5 Å) in the saccharide unit with the unbounded O4*^a^* atom and *chair →twisted boat* (called *type 2* here, for relaxed structure O1*^g^*O1*^a^*≈5.1 Å) in the latter unit with the O1*^a^* oxygen atom in the terminal –OH group. For forces >0.075 au the glycosidic linkage between the galacturonic acid units in the dimer was broken. The stretched and corresponding relaxed dimer structures for the given force values are presented in Figure 6.

Similarly to the galacturonic acid dimer, the stretched/relaxed energy profile presented for the trimer (Figure 3c) could also be conveniently divided into several sections. Rapid energy changes are caused by structural shifts in the molecules and seen in the stretched/relaxed energy profiles. However, this does not always mean a conformational transition. Such a situation took place in this case.

Although several regions could be distinguished in the stretched/relaxed energy profile up to *f* = 0.0495 au (4.08 nN) (Figure 3c), there were no conformational changes in the galacturonic acid units. The distances between distinctive oxygen atoms (i.e. *relaxed* oligomer unit ‘lengths’) were about 4.5 Å and corresponded to the chair conformations (^4^C_1_). Although the differences in the ‘molecule lengths’ (i.e. distances O4*^a^*O1*^a^*) did not exceed 0.2 Å, the spatial arrangements of the saccharide units were modified. The characteristic glycoside dihedral angles (ψ and φ – defined in Figure 2) in relaxed structures varied considerably (ψ_1_ = −40.3° and φ_1_ = +5.6° for *f*≤0.038 au, 3.13 nN, versus ψ_1_ = +25.3° and φ_1_ = 38.9° for 0.0485≤*f*≤0.0495 au, 4.00≤*f*≤4.08 nN). Applying forces in the range of 0.0500–0.0525 au (4.12–4.33 nN) caused the conformational transition *chair→twisted chair type 1* in the saccharide units with the O4*^a^* oxygen atom (for the relaxed structure O4*^a^*O1*^g^*≈4.3 Å). The remaining two oligomer units were not affected. In the next small range of the external stretching forces (0.05275≤ *f*≤0.0535 au, 4.35≤*f*≤4.41 nN) there were two conformational transitions (*chair →twisted chair type 1*) in the O4*^a^*O1*^g^* and O1*^g^*O2*^g^* oligosaccharide units. The last unit remained unchanged. In the region with forces >0.05375 au there were three conformational transitions involving all the oligomer units: *chair→inverted chair* (O4*^a^*O1*^g^* unit), *chair→twisted boat type 2* (O1*^g^*O2*^g^* unit) and *chair→twisted boat type* 1 (O2*^g^*O1*^a^* unit). Applying forces >0.075 au caused the glycoside bonds to break. The selected stretched and corresponding relaxed trimer structures are depicted in Figure S1.

**Figure 7:**
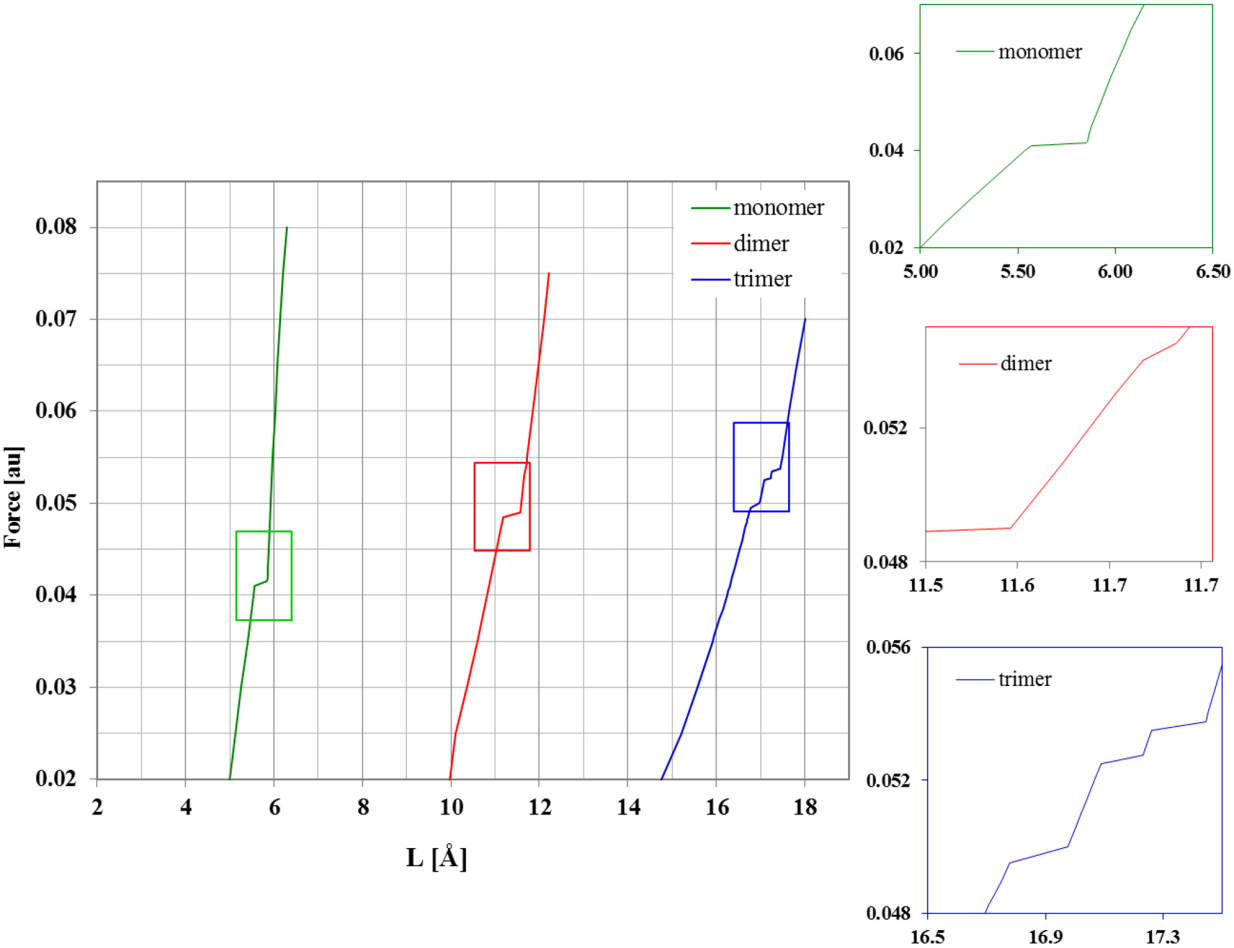
Force extensions curves. Theoretical force extension curves for mono-, di and trimer structures.

A typical property studied by the AFM experiment is the elongation of the polymer as a function of the applied force. The experimental force-extension curves obtained for single-pectin molecules by Marszałek et al. [26] revealed two plateaux at forces ≈300 and ≈800–900 pN and can be interpreted as increased extensibility of the molecules due to a two-step conformational transition of the glucopyranose ring. The theoretical relations between molecule length and the stretching forces are presented in Figure 7 for monomer, dimer and trimer structures. These force-extension curves revealed, as with the stretched/relaxed energy profile, the possible conformational changes in molecules and could also be divided into separate regions. The first region corresponded to the simple stretching (without any conformational transitions) of the oligomer molecule. In this case the molecule elongated remarkably and reached >60% of its maximum extension, which was consistent with the experimental data. The next crucial region, where the curve had its plateau(x) is marked by the magnified frame in Figure 7. This region corresponded to the conformational change(s) in the molecule. Regarding the specific behavior observed here, only the main features of the theoretical profiles should be noted. The EGO method predicted only one plateau for the monomer structure corresponding to the direct ^4^C_1_→^1^C_4_ flip. For the dimer structure there were two plateaux at forces 0.0485 and 0.054 au (4.00 and 4.45 nN), but the second one was very narrow (ΔL ≈0.05 Å). In the case of the trimer, three plateaux were revealed at forces 0.049, 0.0525 and 0.0535 au. The types of possible transitions related to these plateaux in the oligomers considered were described in the previous sections. The last region of the theoretical force-extension profile corresponded to the stretching of the molecules considered after the conformational changes had taken place. For the maximum (0.07 au) force used in the computations, the length of a molecule increased for the dimer by 4 Å, and by 5 Å for the trimer corresponding to 30 and 40% of the lengths compared to the initial structures of the oligomers considered. The use of forces >0.07 and >0.075 au caused the glycosidic bond to break in the case of the trimer and dimer, respectively.

### Tetramer, pentamer and hexamer oligomer structures under external stretching force

For these oligomers EGO calculations were performed with only one value of the external stretching force, namely 0.07 au. The distances between the distinctive oxygen atoms (O4*^a^*, O1*^a^* and O*^g^*) of the *stretched* (stressed) and corresponding *relaxed* tetramer, pentamer and hexamer molecular structures are collected in Table 3 together with analog data for monomer, dimer and trimer.

**Table 3:** The distances between distinctive oxygen atoms (O4*^a^*, O1*^a^* and O*^g^*) of the *stretched* and corresponding *relaxed* α-D-galacturonic acid oligomer molecules obtained for the selected force value *f = 0.07* au (5.77 nN).

	O4^a^O1*^g^* *O1^g^O2^g^ *O2*^g^*O3*^g^* *O3*^g^*O4*^g^* *O4*^g^*O5*^g^* *O5*^g^*O1*^a^* *L*
	[Å]						
*initial structure*							
monomer	4.525*^c^*						4.525
dimer	4.532*^c^*	4.513*^c^*					8.825
trimer	4.532*^c^*	4.536*^c^*	4.633*^c^*				13.399
tetramer	4.478*^c^*	4.501*^c^*	4.506*^c^*	4.619*^c^*			17.019
pentamer	4.479*^c^*	4.502*^c^*	4.495*^c^*	4.507*^c^*	4.619*^c^*		20.856
hexamer	4.486*^c^*	4.518*^c^*	4.501*^c^*	4.493*^c^*	4.503*^c^*	4.618*^c^*	25.329
monomer (anion)*	4.559						4.559
dimer (anion)**	4.530	4.475					8.617
*stretched structure f = 0.07 au*							
monomer	6.146						6.146
dimer	6.091	6.046					12.111
trimer	6.087	5.946	6.043				18.014
tetramer	6.094	5.979	5.970	6.066			23.994
pentamer	6.094	5.978	5.971	5.968	6.066		29.920
hexamer	6.090	5.964	5.971	5.968	5.968	6.066	35.830
monomer (anion)*	6.043						6.043
dimer (anion)**	5.721	5.797					11.498
*relaxed structure*							
monomer	5.527*^ic^*						5.527
dimer	5.511*^ic^*	5.065*^b2^*					10.093
trimer	5.512*^ic^*	5.128*^b2^*	4.310*^b1^* *				12.910
tetramer	5.496*^ic^*	4.383*^b1^*	4.329*^b1^*	5.175*^b2^*			15.221
pentamer	5.500*^ic^*	4.365*^b1^*	4.393*^b1^*	4.381*^b1^*	5.123*^b2^*		16.735
hexamer	5.517*^ic^*	5.079*^b2^*	4.340*^b1^*	4.389*^b1^*	4.376*^b1^*	5.123*^b2^*	20.964
monomer (anion)*	5.532*^ic^*						5.532
dimer (anion)**	5.483*^ic^*	5.040*^b2^*					10.318

c – chair conformation (^4^C_1_).

b1– twisted boat conformation (1).

b1* - twisted boat conformation (1*).

b2– twisted boat conformation (2).

ic –inverted chair (^1^C_4_).

O^g^* = O1*^a^* – for a terminal monomer unit.

*for *f = *0.60 au (4.94 nN).

**for *f* = 0.45 au (3.71 nN).

The total elongation of the oligomer molecules resulting from the external force was approximately 1.5–2 Å per monomer unit (∼40% increase in molecule length compared to the initial structure). The starting, stretched and corresponding relaxed molecular structures of the oligosaccharides considered are presented in Figures S2–S4 for tetramer, pentamer and hexamer.

The stretching of progressively longer oligomers allowed for an indication of the representative oligomeric structure (minimum size of α-D-galacturonic acid oligomer). In the tetrameric (or even trimeric) structure of α-D-galacturonic acid all characteristic enforced conformational transition occurred. Thus, it seems that the simplest representative (i.e. suitable for the theoretical description of nano-mechanical properties) oligomer contained three/four units of α-D-galacturonic acid.

### Ionization of carboxylic groups in (poly)galacturonic acid

Polygalacturonic acid chains contain a large number of ionizable carboxylic groups. In this paper we present the detailed results for six galacturonic acid oligomers. For clarity, we limited our discussion up to neutral (non-ionized) and non-esterified molecules. However, under biological conditions these effects can play important roles and may significantly influence the conformation and flexibility of linkages between the galacturonic residues. Therefore some test calculations for anionic monomeric and dimeric structures were carried out. The results for selected forces are presented in Table 3 together with data for the subsequent nonionic saccharide oligomers. The distances between the distinctive oxygen atoms of the stretched and corresponding relaxed anionic structures (and therefore the types of conformational transition in saccharide rings) were consistent with the results obtained for neutral molecules.

It should be emphasized that even in the case of the trimer there were several possible ways of ionization/esterification of the carboxylic groups. In nature (ionizable in solution) carboxyl groups of galacturonic acid in pectin can often be esterified (usually with methanol).The proportion of carboxyl groups esterified defines the so called degree of methyl esterification (or methoxylation). If >50% of the carboxyl groups are methylated the pectins are called high-methoxy pectins - such pectins are usually present in nature. Pectins with <50% of carboxyl groups esterified are called low-methoxy pectins - such pectins can generally be obtained by, for example, controlled acid de-esterification of high-metoxy pectins. The detailed explanation of the ionization and degree and pattern of esterification effects on the conformational properties of considered structures requires further study and will be investigated in the future.

### The course and types of structural changes

**Figure 8:**
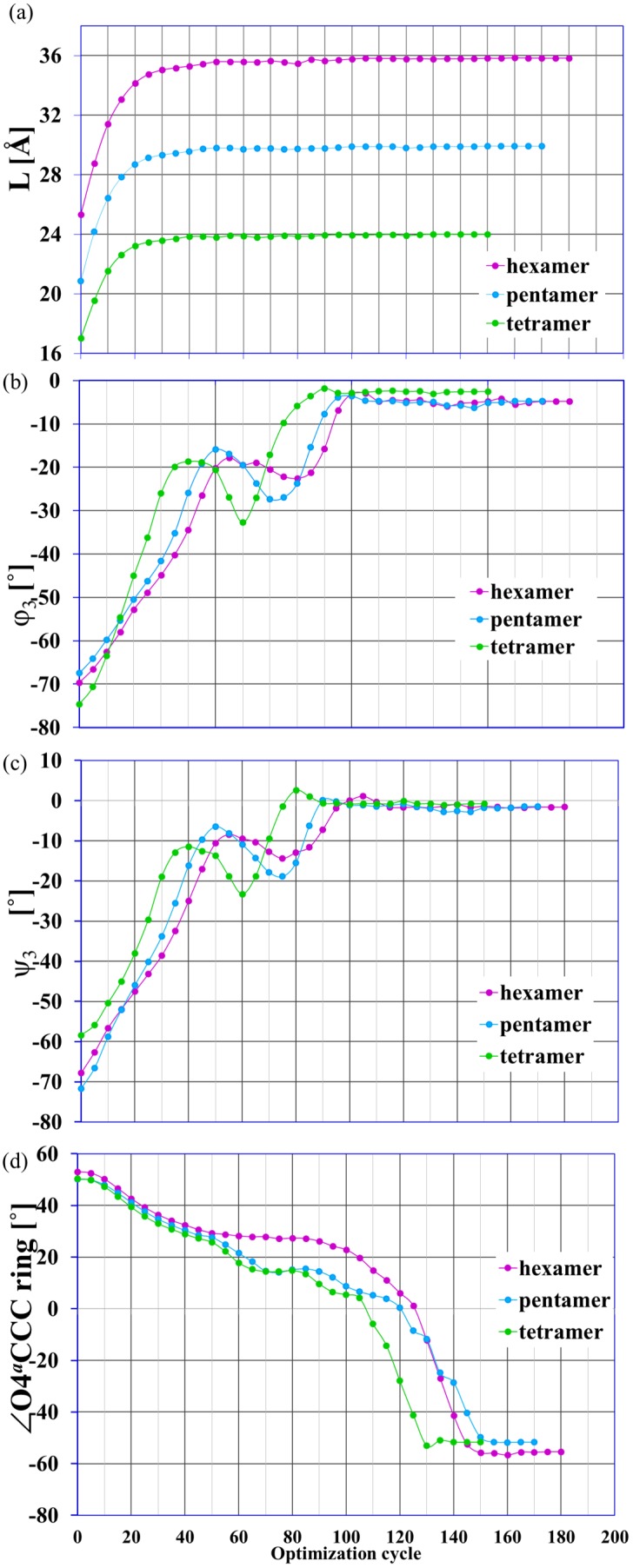
Structural parameters as a function of the optimization cycle. The selected structural parameters as a function of the optimization cycle number; the enforced optimization in the presence of external force *f = *0.07 au applied to terminal oxygen atoms O4*^a^* and O1*^a^*; a) L = O4*^a^*O1*^a^* distance, b) angles φ_3_ and c) ψ_3_ defined similarly as in Figure 1, d) ∠O4*^a^*CCC is the dihedral angle in the ring of the terminal monomer units with the unbounded O4*^a^* atom.

The EGO procedure simulates the structural changes in the molecule induced by an external force. How does this enforced reaction occur? In the first stage of the enforced geometry optimizations (for the given stretching force *f = 0.07* au), the drastic elongation of all the oligomer chains took place. In fact, the molecules almost reached their final lengths (Figure 8a). This increase in length of the considered oligomers was not associated with any conformational transitions, but was connected to the mutual rotation of the oligosaccharide units around the glycosidic linkages.

It can be seen in the changes of the characteristic values of the dihedral glycosidic angles (Figure 8b and c). In the next step the chain elongation and monomer units’ rotations were significantly less noticeable and there were conformational changes in the monomer units of the considered oligomers. These changes were manifest by rapid structural shifts in glucopyranose rings (e.g. Figure 8d). For more details please see the EGO simulation for hexamer oligomer in Movie S1.

The type of conformational transitions generally depends on the unit position in the oligosaccharide chain. The transition *chair → inverted chair* occurred in the case of the terminal monomer units with the unbound O4*^a^* atom. For the remaining units the transitions *chair→‘boat-like’ conformation(s)* could be observed. It should be noted that the relaxation of the stretched oligosaccharides yielded the twisted boat conformation type 2 (b2) (with the relaxed monomer ‘length’ of about 5.1 Å) of the terminal monomer units with the O1*^a^* atom. In the case of all other (i.e., internal) units, the conformational flip *chair → twisted boat b1* took place with the relaxed monomer ‘length’ of about 4.1 Å.

### Characterization of ring conformations

The EGO method predicts three possible permanent conformational changes in the considered α-D-galacturonic acid oligomers. These conformations can be identified in different ways [29], [30], [31]. Up to now we have used only the oligosaccharide unit ‘length’ of the relaxed oligomers and we have called the boat-like conformations *type 1* or *type 2*. Complete conformational identification was not one of the main goals of this study; however, the endocyclic dihedral angles for example can be used to describe the ring pucker in the resulting oligosaccharide units [30]. This procedure allowed us to determine a particular pucker as the linear combination of ideal canonical conformations (^1^C_4_, ^1,4^B and ^O^S_2_) and express it in terms of the closest matching chair, boat and twist boat conformations.

**Figure 9:**
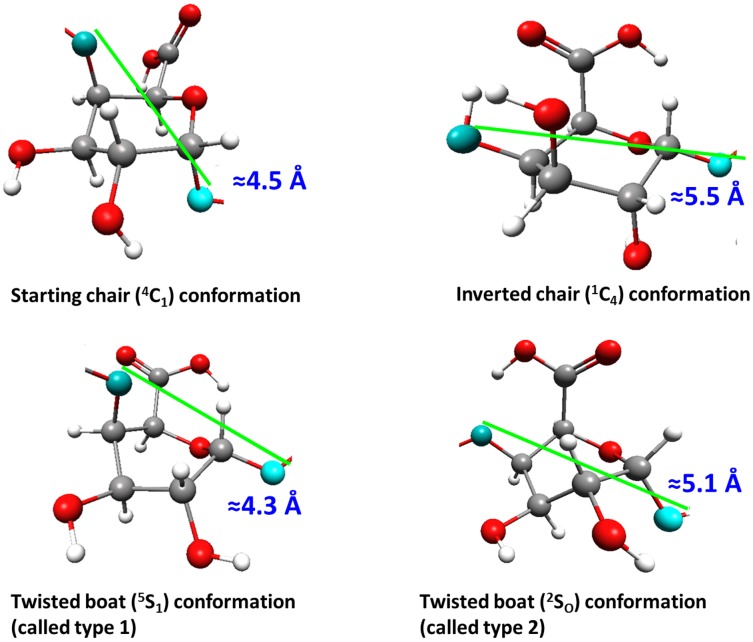
Possible monomer conformations after the EGO procedure. Possible conformations of monomer units in *relaxed* oligomer structures obtained after the EGO procedure.

The analysis of the endocyclic torsion angle gave the best matching conformations of particular saccharide units (for details please see Figure S5 and Table S4). The conformations labeled, until now, *type 1* and *type 2* can be identified as ^5^S_1_ and ^2^S_O_, respectively (Figure 9). It should also be emphasized that the position of the monosaccharide ring in the (poly)/oligomeric structure of α-D-galacturonic acid determines the type of the enforced conformational transitions. For all terminal monomer units with the O4*^a^* unbound atom the (full) conformational flip ^4^C_1_→^1^C_4_ took place. In the second type of terminal units (with the O1*^a^* unbound atom) the staring ^4^C_1_ conformer generally converted into the ^2^S_O_ form. For all remaining units of α-D-galacturonic acid located in the center of oligomeric molecules the enforced transition ^4^C_1_→^5^S_1_ was observed.

## Conclusions

The AFM allows for the investigation of enforced conformational changes in carbohydrates. These structural transitions may affect many biological processes. However, despite their importance, the mechanisms that underlie the mentioned structural shifts are still of interest and not always sufficiently understood. The molecular simulations of AFM experiments may help to explain and describe the way that these changes occur.

The EGO description of the force-driven conformational changes in α-D-galacturonic acid monomer showed that *the mechanism* of direct inversion *chair→inverted chair* in the pyranose ring indeed involved an intermediate transition into a twisted boat conformer. It indicates that the EGO approach properly reproduces the experimental trend.

Additionally, it was also shown that the position of the monosaccharide ring in the (poly)/oligomeric structure of α-D-galacturonic acid may have had significant effects on the type of force-driven conformational/structural changes. The enforced conformational transition ^4^C_1_→^1^C_4_ occurred in all terminal monomer units with the O4*^a^* unbound atom, while generally for the second type of terminal units (with the O1*^a^* unbound atom) the conversion^ 4^C_1_→^2^S_O_ of pyranose ring took place. For all remaining units of α-D-galacturonic acid located in the center of oligomeric molecules the enforced transition ^4^C_1_→^5^S_1_ was observed.

The results discussed above also allow for the identification of the representative oligomeric structure (minimum size of α-D-galacturonic acid oligomer) for the theoretical investigation of the nano-mechanical properties of pectic acid, especially at the quantum-mechanical level. The tetrameric (or even trimeric) structure of α-D-galacturonic acid seems to be appropriate for the theoretical description of the enforced conformational changes in this molecular system.

Finally, it should be noted that the theoretical molecular model presented in this work is adequate for linear and unbranched homogalacturonans. The development of a theoretical model for the molecules of branched pectins from plant cell walls requires further study.

## Supporting Information

Figure S1 **Stretched and relaxed trimer structures.** The selected stretched and corresponding *relaxed* trimer structures with marked oligomer unit lengths.
(TIF)

Figure S2 **Stretched and relaxed tetramer structures.** The stretched and corresponding relaxed tetramer structures with marked oligomer unit lengths.
(TIF)

Figure S3 **Stretched and relaxed pentamer structures.** The stretched and corresponding relaxed pentamer structures with marked oligomer unit lengths.
(TIF)

Figure S4 **Stretched and relaxed heksamer structures.** The stretched and corresponding relaxed hexamer structures with marked oligomer unit lengths.
(TIF)

Figure S5 **Torsion angles.** The definition of endocyclic torsion angles.
(TIF)

Table S1 **Distances between oxygen atoms of the stretched and relaxed α-D-galacturonic acid monomer.** Distances between distinctive oxygen atoms (O4*^a^*, O1*^a^*) of the stretched and corresponding relaxed α-D-galacturonic acid monomer structures obtained as a result of external forces *f* working on O1*^a^* and O4*^a^* atoms.
(DOCX)

Table S2 **Distances between oxygen atoms of the stretched and relaxed α-D-galacturonic acid dimer.** Distances between distinctive oxygen atoms (O4*^a^*, O1*^a^* and O*^g^*) of the stretched and relaxed α-D-galacturonic acid dimer structures obtained as a result of external forces *f* working on O1*^a^* and O4*^a^* atoms.
(DOCX)

Table S3 **Distances between oxygen atoms of the stretched and relaxed α-D-galacturonic acid trimer.** Distances between distinctive oxygen atoms (O4*^a^*, O1*^a^* and O*^g^*) of the stretched and relaxed α-D-galacturonic acid trimer structures obtained as a result of external forces *f* working on O1*^a^* and O4*^a^* atoms.
(DOCX)

Table S4 **The characterization of six-membered ring conformation in the saccharide units of considered molecules.**
(DOCX)

Movie S1 **EGO simulation for hexamer.**
(FLV)
