# Supplementary figures and images for: Simulation of Force Spectroscopy Experiments on Galacturonic Acid Oligomers
Source: PLoS One. 2014 Sep 17;9(9):e107896. doi: 10.1371/journal.pone.0107896 (PMC4168238; doi:10.1371/journal.pone.0107896)

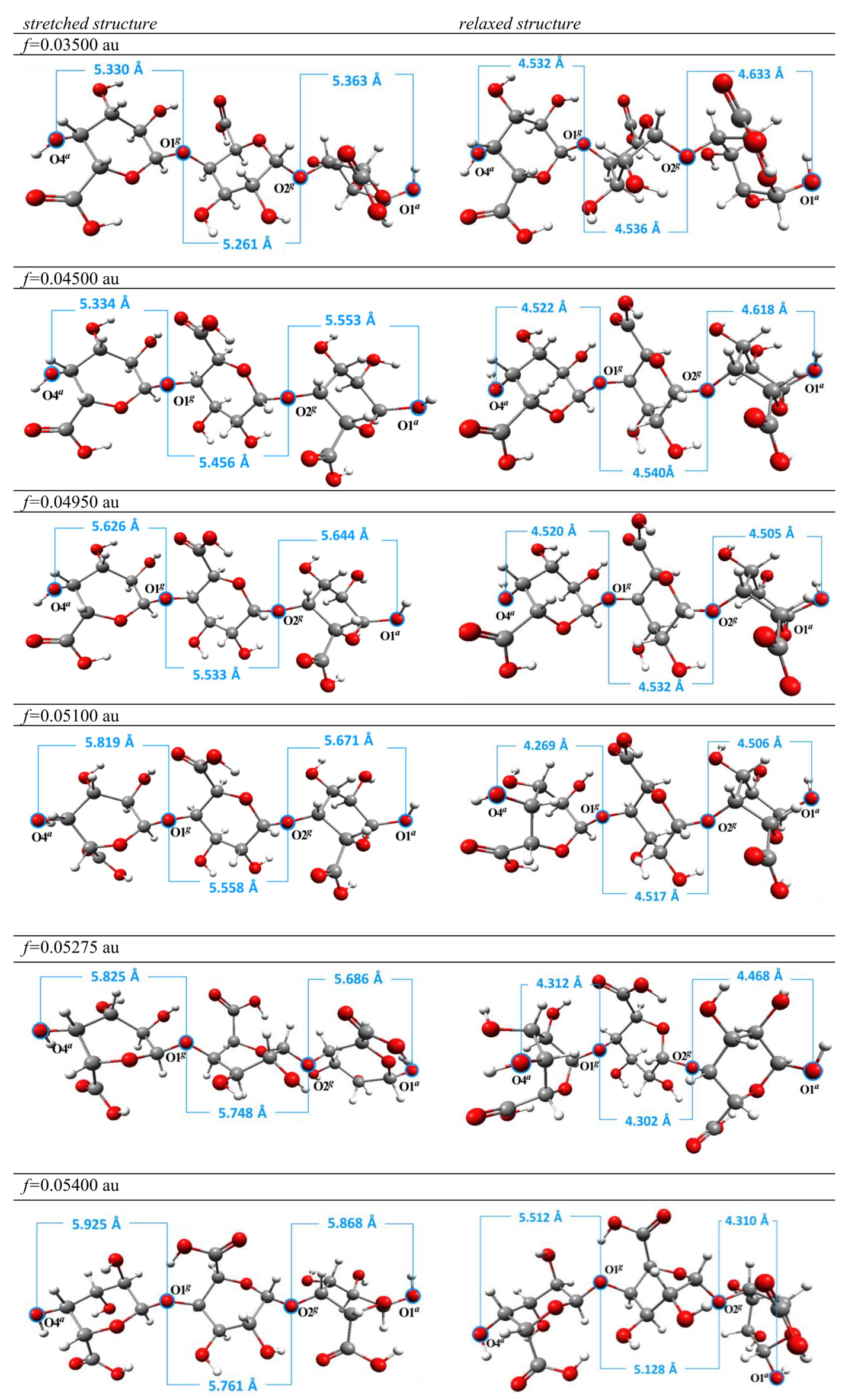

Supplement: Figure S1 — Stretched and relaxed trimer structures. The selected stretched and corresponding relaxed trimer structures with marked oligomer unit lengths. (TIF) [file pone.0107896.s001.tif]

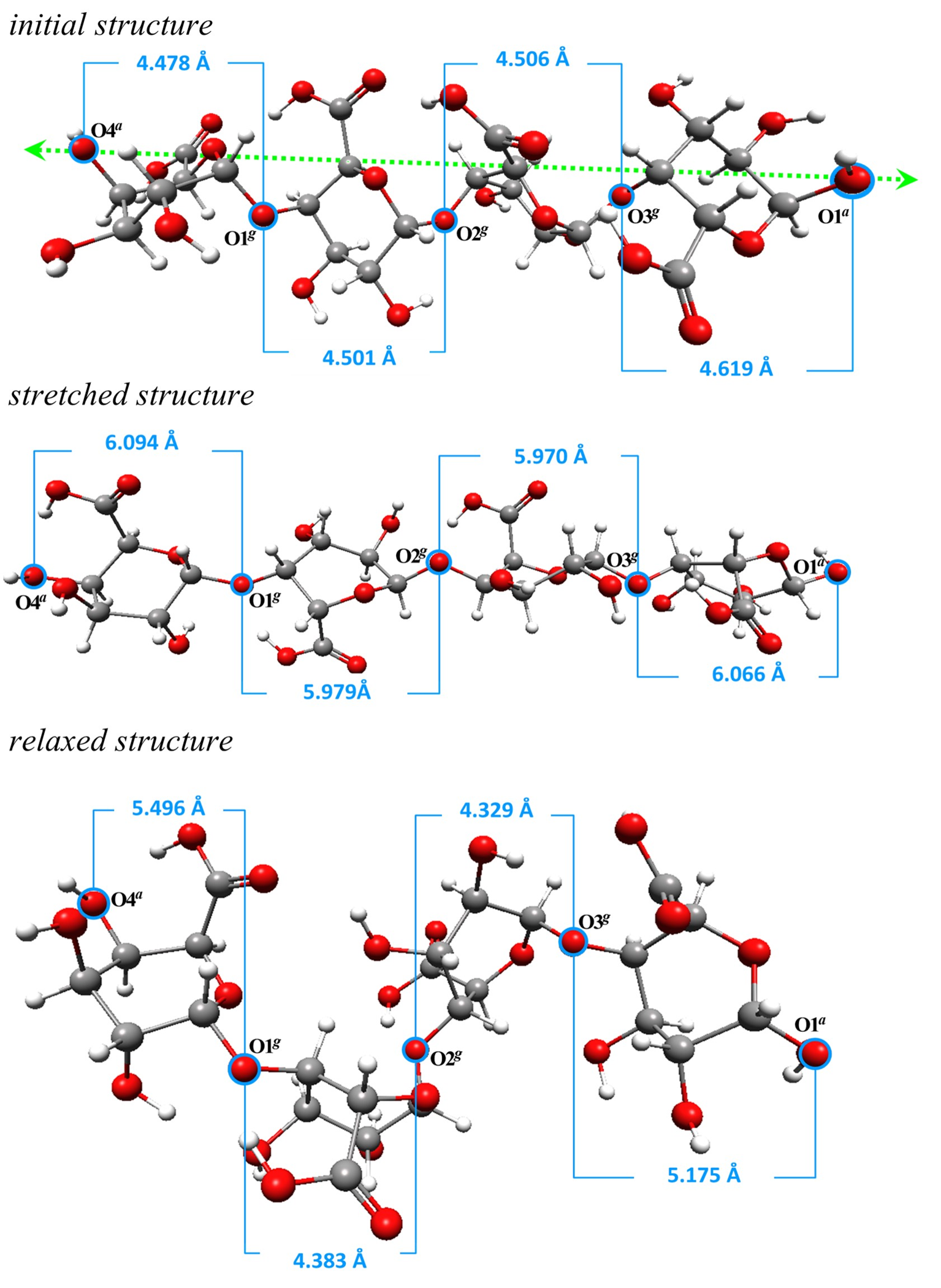

Supplement: Figure S2 — Stretched and relaxed tetramer structures. The stretched and corresponding relaxed tetramer structures with marked oligomer unit lengths. (TIF) [file pone.0107896.s002.tif]

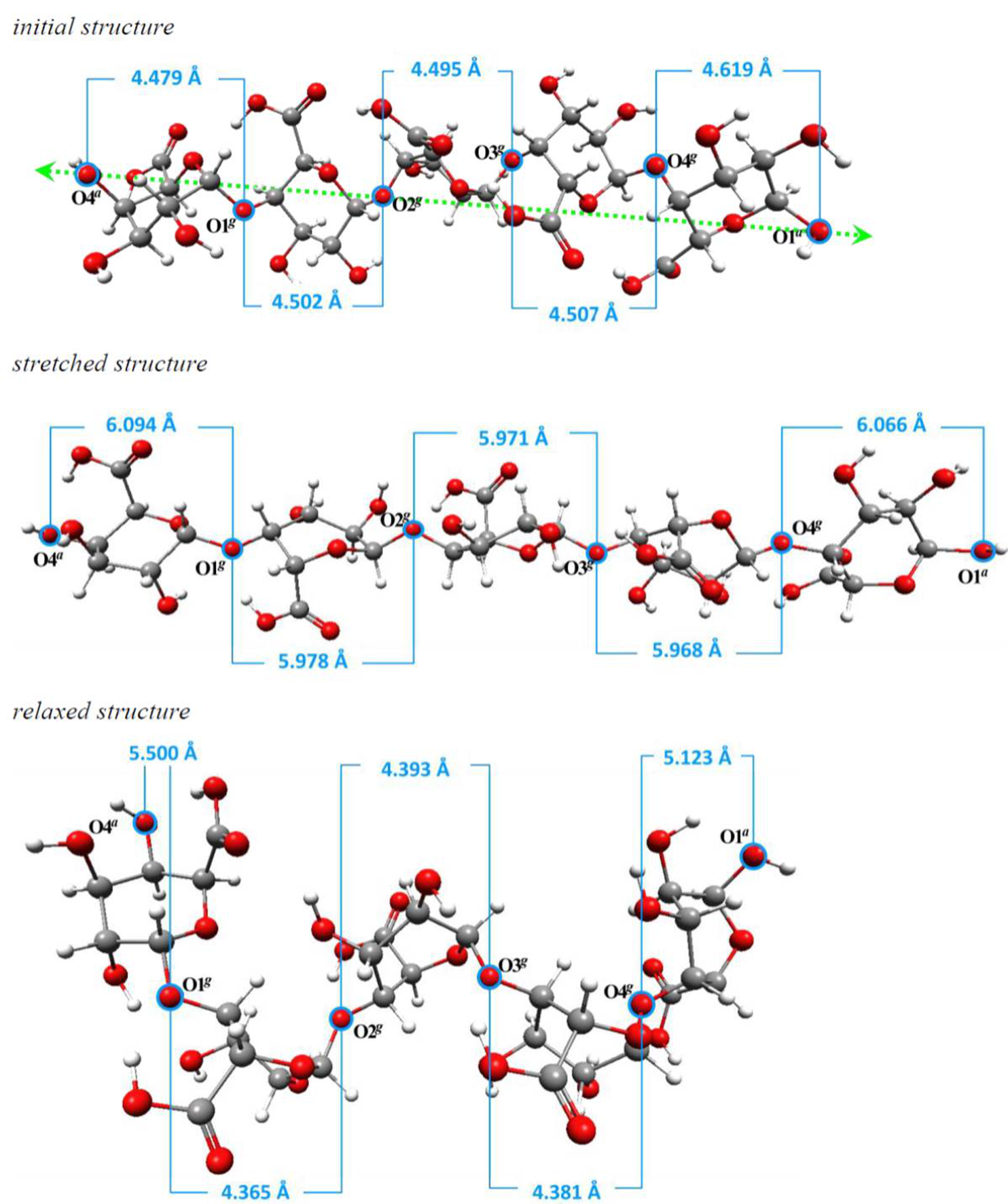

Supplement: Figure S3 — Stretched and relaxed pentamer structures. The stretched and corresponding relaxed pentamer structures with marked oligomer unit lengths. (TIF) [file pone.0107896.s003.tif]

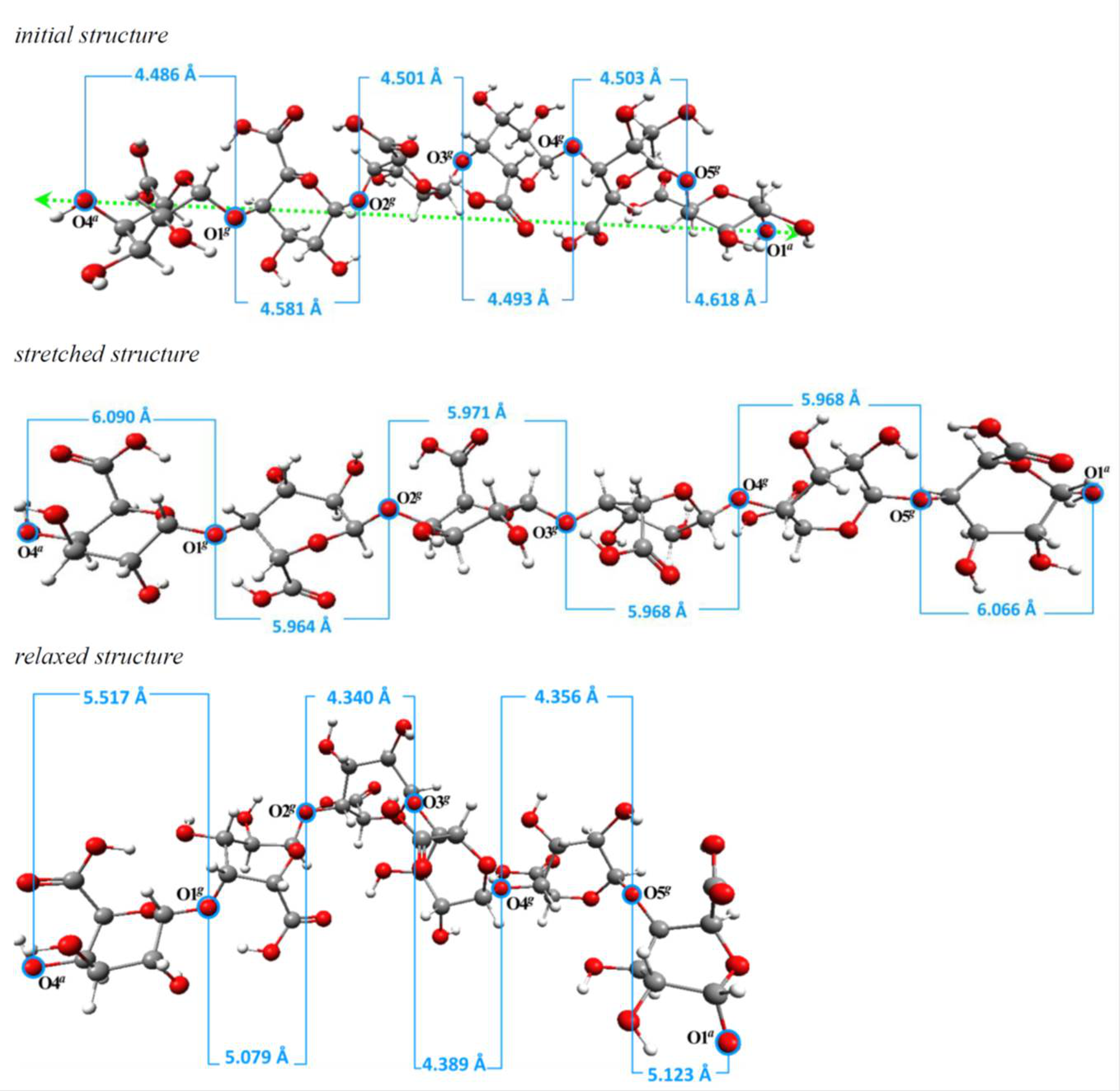

Supplement: Figure S4 — Stretched and relaxed heksamer structures. The stretched and corresponding relaxed hexamer structures with marked oligomer unit lengths. (TIF) [file pone.0107896.s004.tif]

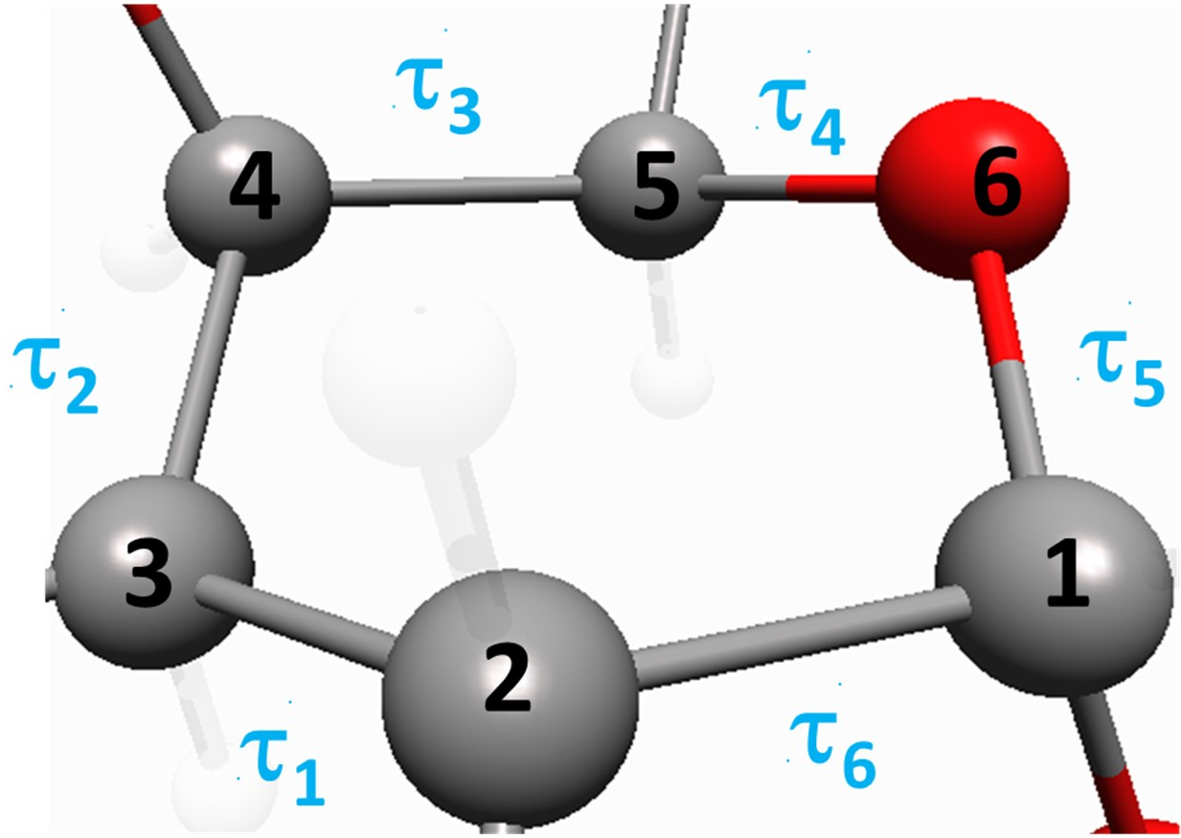

Supplement: Figure S5 — Torsion angles. The definition of endocyclic torsion angles. (TIF) [file pone.0107896.s005.tif]
