# Supplementary material for: Simulation of Force Spectroscopy Experiments on Galacturonic Acid Oligomers
Source: PLoS One. 2014 Sep 17;9(9):e107896. doi: 10.1371/journal.pone.0107896 (PMC4168238; doi:10.1371/journal.pone.0107896)
Supplement: Table S1 — Distances between oxygen atoms of the stretched and relaxed α-D-galacturonic acid monomer. Distances between distinctive oxygen atoms (O4a, O1a) of the stretched and corresponding relaxed α-D-galacturonic acid monomer structures obtained as a result of external forces f working on O1a and O4a atoms. (DOCX) [file pone.0107896.s006.docx]

SUPPORTING TABLE S1 for

Simulation of force spectroscopy experiments on galacturonic acid oligomers

Justyna Cybulska, Agnieszka Brzyska, Artur Zdunek, and Krzysztof Woliński

**Table S1.** Distances between distinctive oxygen atoms (O4*^a^*, O1*^a^*) of the *stretched* and corresponding *relaxed* α-D-galacturonic acid monomer structures obtained as a result of external forces *f* working on O1*^a^* and O4*^a^* atoms.

|  | *Stretched structure* |  | *Relaxed structure* | | |
| --- | --- | --- | --- | --- | --- |
| *f* | **O4*^a^*O1^a^** |  | **O4*^a^*O1^a^** | | |
| **[au]** | **Å** |  | **Å** | | |
|  | *no conformation changes* | | | | |
| **0.0000** | 4.524*^c^* |  |  | | |
| **0.0200** | 4.997 |  | 4.524*^c^* | | |
| **0.0250** | 5.127 |  | 4.524*^c^* | | |
| **0.0300** | 5.259 |  | 4.524*^c^* | | |
| **0.0350** | 5.398 |  | 4.524*^c^* | | |
| **0.0400** | 5.539 |  | 4.525*^c^* | | |
|  | *chair →inverted chair* | | | | |
| **0.0410** | 5.568 |  | 5.527 *^ic^* |  |  |
| **0.0420** | 5.858 |  | 5.527 *^ic^* |  |  |
| **0.0430** | 5.862 |  | 5.527 *^ic^* |  |  |
| **0.0450** | 5.880 |  | 5.527 *^ic^* |  |  |
| **0.0500** | 5.927 |  | 5.527 *^ic^* |  |  |
| **0.0550** | 5.976 |  | 5.527 *^ic^* |  |  |
| **0.0600** | 6.028 |  | 5.527 *^ic^* |  |  |
| **0.0650** | 6.085 |  | 5.527 *^ic^* |  |  |
| **0.0700** | 6.146 |  | 5.527 *^ic^* |  |  |
| **0.0750** | 6.215 |  | 5.527 *^ic^* |  |  |
| **0.0800** | 6.296 |  | 5.527 *^ic^* |  |  |
| *ic –inverted chair* (^1^C_4_) *c* – *chair conformation* (^4^C_1_) | | | | |  |
