# Supplementary material for: Simulation of Force Spectroscopy Experiments on Galacturonic Acid Oligomers
Source: PLoS One. 2014 Sep 17;9(9):e107896. doi: 10.1371/journal.pone.0107896 (PMC4168238; doi:10.1371/journal.pone.0107896)
Supplement: Table S2 — Distances between oxygen atoms of the stretched and relaxed α-D-galacturonic acid dimer. Distances between distinctive oxygen atoms (O4a, O1a and Og) of the stretched and relaxed α-D-galacturonic acid dimer structures obtained as a result of external forces f working on O1a and O4a atoms. (DOCX) [file pone.0107896.s007.docx]

SUPPORTING TABLE S2 for

Simulation of force spectroscopy experiments on galacturonic acid oligomers

Justyna Cybulska, Agnieszka Brzyska, Artur Zdunek, and Krzysztof Woliński

**Table S2.** Distance between distinctive oxygen atoms (O4*^a^*, O1*^a^* and O*^g^*) of the stretched and *relaxed* α-D-galacturonic acid dimer structures obtained as a result of external forces *f* working on O1*^a^* and O4*^a^* atoms.

|  | *Stretched structure* | | |  | *Relaxed structure* | | |
| --- | --- | --- | --- | --- | --- | --- | --- |
| ***F*** | **O4*^a^*O1*^g^*** | **O1*^g^*O1*^a^*** | **O4*^a^*O1*^a^*** |  | **O4*^a^*O1*^g^*** | **O1*^g^*O1*^a^*** | **O4*^a^*O1*^a^*** |
| **[au]** | **[Å]** | | |  | **[Å]** | | |
|  | *no conformation changes (stretching)* | | | | | | |
| **0.000** | 4.532 *^c^* | 4.513 *^c^* | 8.825 |  |  |  |  |
| **0.020** | 4.938 | 4.927 | 9.974 |  | 4.532 *^c^* | 4.513 *^c^* | 8.825 |
| **0.025** | 5.090 | 5.053 | 10.107 |  | 4.532 *^c^* | 4.513 *^c^* | 8.825 |
| **0.030** | 5.216 | 5.171 | 10.361 |  | 4.532 *^c^* | 4.513 *^c^* | 8.825 |
| **0.035** | 5.340 | 5.279 | 10.605 |  | 4.532 *^c^* | 4.513 *^c^* | 8.828 |
| **0.040** | 5.447 | 5.383 | 10.820 |  | 4.532 *^c^* | 4.513 *^c^* | 8.829 |
| **0.045** | 5.548 | 5.487 | 11.028 |  | 4.532 *^c^* | 4.513 *^c^* | 8.827 |
| **0.047** | 5.590 | 5.530 | 11.113 |  | 4.532 *^c^* | 4.513 *^c^* | 8.829 |
| **0.048** | 5.612 | 5.556 | 11.161 |  | 4.533 *^c^* | 4.514 *^c^* | 8.829 |
|  | *chair/chair→ twisted boat1/twisted boat1* | | | | | | |
| **0.049** | 5.840 | 5.762 | 11.574 |  | 4.327*^b1^* | 4.367 *^b1^* | 6.443 |
| **0.050** | 5.851 | 5.774 | 11.597 |  | 4.323 *^b1^* | 4.368 *^b1^* | 6.441 |
| **0.051** | 5.861 | 5.786 | 11.620 |  | 4.326 *^b1^* | 4.367 *^b1^* | 6.439 |
| **0.053** | 5.882 | 5.881 | 11.665 |  | 4.321 *^b1^* | 4.368 *^b1^* | 6.440 |
| **0.054** | 5.893 | 5.823 | 11.689 |  | 4.325 *^b1^* | 4.367 *^b1^* | 6.442 |
|  | *chair/chair→ inverted chair/twisted boat2* | | | | | | |
| **0.055** | 5.930 | 5.833 | 11.730 |  | 5.512 *^ic^* | 5.066 *^b2^* | 10.093 |
| **0.060** | 5.980 | 5.899 | 11.848 |  | 5.512 *^ic^* | 5.066 *^b2^* | 10.093 |
| **0.065** | 6.033 | 5.969 | 11.974 |  | 5.512 *^ic^* | 5.066 *^b2^* | 10.093 |
| **0.070** | 6.091 | 6.046 | 12.111 |  | 5.511 *^ic^* | 5.065 *^b2^* | 10.093 |
| **0.075** | 6.155 | 6.135 | 12.226 |  | 5.512 *^ic^* | 5.065 *^b2^* | 10.094 |
| *c* – *chair conformation* (^4^C_1_),  *b1* – *twisted boat conformation(1),*  *b2- twisted boat conformation (2),*  *ic –inverted chair* (^1^C_4_) | | | | | | | |
