# Supplementary material for: Simulation of Force Spectroscopy Experiments on Galacturonic Acid Oligomers
Source: PLoS One. 2014 Sep 17;9(9):e107896. doi: 10.1371/journal.pone.0107896 (PMC4168238; doi:10.1371/journal.pone.0107896)
Supplement: Table S3 — Distances between oxygen atoms of the stretched and relaxed α-D-galacturonic acid trimer. Distances between distinctive oxygen atoms (O4a, O1a and Og) of the stretched and relaxed α-D-galacturonic acid trimer structures obtained as a result of external forces f working on O1a and O4a atoms. (DOCX) [file pone.0107896.s008.docx]

SUPPORTING TABLE S3 for

Simulation of force spectroscopy experiments on galacturonic acid oligomers

Justyna Cybulska, Agnieszka Brzyska, Artur Zdunek, and Krzysztof Woliński

**Table S3.** Distances between distinctive oxygen atoms (O4*^a^*, O1*^a^* and O*^g^*) of the stretched and *relaxed* α-D-galacturonic acid trimer structures obtained as a result of external forces *f* working on O1*^a^* and O4*^a^* atoms.

|  | *Stretched structure* | | | |  | *Relaxed structure* | | | | |
| --- | --- | --- | --- | --- | --- | --- | --- | --- | --- | --- |
| ***f*** | **O4*^a^*O1*^g^*** | **O1*^g^*O2*^g^*** | **O2*^g^*O1*^a^*** | **O4*^a^*O1*^a^*** |  | **O4*^a^*O1*^g^*** | **O1*^g^*O2*^g^*** | **O2*^g^*O1*^a^*** | **O4*^a^*O1*^a^*** | |
| **[au]** | **[Å]** | | | |  | **[Å]** | | | | |
|  | *no conformation changes* | | | | | | | | |  |
| **0.00000** | 4.532 | 4.536 | 4.633 | 13.399 |  | 4.532 *^c^* | 4.536 *^c^* | 4.633 *^c^* | 13.399 | |
| **0.02000** | 4.933 | 4.906 | 5.057 | 14.758 |  | 4.532 *^c^* | 4.536 *^c^* | 4.633 *^c^* | 13.398 | |
| **0.02500** | 5.082 | 5.034 | 5.160 | 15.200 |  | 4.532 *^c^* | 4.536 *^c^* | 4.633 *^c^* | 13.399 | |
| **0.03000** | 5.214 | 5.155 | 5.264 | 15.585 |  | 4.532 *^c^* | 4.536 *^c^* | 4.633 *^c^* | 13.399 | |
| **0.03500** | 5.330 | 5.261 | 5.363 | 15.922 |  | 4.532 *^c^* | 4.536 *^c^* | 4.633 *^c^* | 13.399 | |
| **0.03550** | 5.373 | 5.272 | 5.413 | 15.954 |  | 4.532 *^c^* | 4.536 *^c^* | 4.633 *^c^* | 13.399 | |
| **0.03750** | 5.385 | 5.323 | 5.412 | 16.082 |  | 4.532 *^c^* | 4.536 *^c^* | 4.633 *^c^* | 13.399 | |
| **0.03800** | 5.386 | 5.323 | 5.422 | 16.115 |  | 4.532 *^c^* | 4.536 *^c^* | 4.633 *^c^* | 13.399 | |
|  |  |  |  |  |  |  |  |  |  | |
| **0.03850** | 5.409 | 5.333 | 5.431 | 16.149 |  | 4.543 *^c^* | 4.528 *^c^* | 4.639 *^c^* | 13.352 | |
| **0.03900** | 5.420 | 5.343 | 5.441 | 16.180 |  | 4.544 *^c^* | 4.528 *^c^* | 4.639 *^c^* | 13.352 | |
| **0.03950** | 5.430 | 5.353 | 5.450 | 16.211 |  | 4.543 *^c^* | 4.528 *^c^* | 4.639 *^c^* | 13.351 | |
| **0.04000** | 5.441 | 5.364 | 5.460 | 16.241 |  | 4.544 *^c^* | 4.528 *^c^* | 4.639 *^c^* | 13.353 | |
| **0.04050** | 5.451 | 5.374 | 5.469 | 16.272 |  | 4.544 *^c^* | 4.528 *^c^* | 4.639 *^c^* | 13.351 | |
|  |  |  |  |  |  |  |  |  |  | |
| **0.04075** | 5.451 | 5.380 | 5.471 | 16.285 |  | 4.522 *^c^* | 4.537 *^c^* | 4.618 *^c^* | 13.516 | |
| **0.04100** | 5.456 | 5.385 | 5.476 | 16.299 |  | 4.522 *^c^* | 4.539 *^c^* | 4.619 *^c^* | 13.516 | |
| **0.04200** | 5.476 | 5.403 | 5.495 | 16.355 |  | 4.521 *^c^* | 4.539 *^c^* | 4.619 *^c^* | 13.516 | |
| **0.04300** | 5.495 | 5.421 | 5.514 | 16.411 |  | 4.521 *^c^* | 4.540 *^c^* | 4.618 *^c^* | 13.517 | |
| **0.04500** | 5.534 | 5.456 | 5.553 | 16.523 |  | 4.522 *^c^* | 4.540 *^c^* | 4.618 *^c^* | 13.517 | |
| **0.04600** | 5.554 | 5.474 | 5.572 | 16.579 |  | 4.520 *^c^* | 4.539 *^c^* | 4.618 *^c^* | 13.515 | |
| **0.04700** | 5.574 | 5.492 | 5.591 | 16.635 |  | 4.521 *^c^* | 4.539 *^c^* | 4.618 *^c^* | 13.517 | |
| **0.04750** | 5.584 | 5.500 | 5.601 | 16.663 |  | 4.523 *^c^* | 4.539 *^c^* | 4.618 *^c^* | 13.516 | |
| **0.04775** | 5.589 | 5.505 | 5.607 | 16.678 |  | 4.521 *^c^* | 4.539 *^c^* | 4.616 *^c^* | 13.517 | |
|  |  |  |  |  |  |  |  |  |  | |
| **0.04800** | 5.594 | 5.509 | 5.613 | 16.693 |  | 4.528 *^c^* | 4.544 *^c^* | 4.618 *^c^* | 13.531 | |
| **0.04825** | 5.599 | 5.514 | 5.617 | 16.707 |  | 4.528 *^c^* | 4.544 *^c^* | 4.618 *^c^* | 13.530 | |
|  |  |  |  |  |  |  |  |  |  | |
| **0.04850** | 5.604 | 5.516 | 5.626 | 16.723 |  | 4.520 *^c^* | 4.532 *^c^* | 4.505 *^c^* | 13.423 | |
| **0.04900** | 5.615 | 5.524 | 5.635 | 16.751 |  | 4.520 *^c^* | 4.532 *^c^* | 4.505 *^c^* | 13.423 | |
| **0.04950** | 5.626 | 5.533 | 5.644 | 16.779 |  | 4.520 *^c^* | 4.532 *^c^* | 4.505 *^c^* | 13.423 | |
|  | *chair/chair/chair → twisted boat1/chair/chair* | | | | | | | | | |
| **0.05000** | 5.693 | 5.561 | 5.661 | 16.974 |  | 4.270 *^b1^* | 4.516 *^c^* | 4.506 *^c^* | 12.436 | |
| **0.05050** | 5.814 | 5.549 | 5.663 | 16.997 |  | 4.270 *^b1^* | 4.517 *^c^* | 4.506 *^c^* | 12.435 | |
| **0.05100** | 5.819 | 5.558 | 5.671 | 17.020 |  | 4.269 *^b1^* | 4.571 *^c^* | 4.506 *^c^* | 12.435 | |
| **0.05150** | 5.830 | 5.576 | 5.688 | 17.065 |  | 4.271 *^b1^* | 4.516 *^c^* | 4.505 *^c^* | 12.438 | |
| **0.05200** | 5.836 | 5.585 | 5.697 | 17.088 |  | 4.271 *^b1^* | 4.517 *^c^* | 4.505 *^c^* | 12.438 | |
|  | *chair/chair/chair → twisted boat1/twisted boat1/chair* | | | | | | | | | |
| **0.05250** | 5.825 | 5.748 | 5.686 | 17.232 |  | 4.312 *^b1^* | 4.302 *^b1^* | 4.468 *^c^* | 9.949 | |
| **0.05275** | 5.827 | 5.751 | 5.691 | 17.242 |  | 4.310 *^b1^* | 4.301 *^b1^* | 4.467 *^c^* | 9.938 | |
| **0.05300** | 5.833 | 5.757 | 5.699 | 17.261 |  | 4.313 *^b1^* | 4.302 *^b1^* | 4.468 *^c^* | 9.949 | |
|  | *chair/chair/chair→ inverted chair/twisted boat2/twisted boat1* | | | | | | | | | |
| **0.05350** | 5.913 | 5.747 | 5.855 | 17.443 |  | 5.512 *^ic^* | 5.123 *^b2^* | 4.311 *^b1^* | 12.907 | |
| **0.05375** | 5.915 | 5.750 | 5.857 | 17.452 |  | 5.512 *^ic^* | 5.127 *^b2^* | 4.311 *^b1^* | 12.908 | |
| **0.05400** | 5.925 | 5.761 | 5.868 | 17.484 |  | 5.512 *^ic^* | 5.128 *^b2^* | 4.310 *^b1^* | 12.911 | |
| **0.05500** | 5.976 | 5.819 | 5.923 | 17.650 |  | 5.512 *^ic^* | 5.128 *^b2^* | 4.309 *^b1^* | 12.914 | |
| **0.06000** | 6.029 | 5.880 | 5.981 | 17.826 |  | 5.512 *^ic^* | 5.129 *^b2^* | 4.309 *^b1^* | 12.916 | |
| **0.06500** | 6.087 | 5.946 | 6.043 | 18.014 |  | 5.512 *^ic^* | 5.128 *^b2^* | 4.310 *^b1^* | 12.910 | |
| **0.07000** | 6.087 | 5.946 | 6.043 | 18.014 |  | 5.512 *^ic^* | 5.128 *^b2^* | 4.310 *^b1^* | 12.910 | |
| *c* – *chair conformation* (^4^C_1_), *b1* – *twisted boat conformation(1), b2- twisted boat conformation (2), ic –inverted chair* (^1^C_4_) | | | | | | | | | | |
