# Supplementary material for: Simulation of Force Spectroscopy Experiments on Galacturonic Acid Oligomers
Source: PLoS One. 2014 Sep 17;9(9):e107896. doi: 10.1371/journal.pone.0107896 (PMC4168238; doi:10.1371/journal.pone.0107896)
Supplement: Table S4 — The characterization of six-membered ring conformation in the saccharide units of considered molecules. (DOCX) [file pone.0107896.s009.docx]

SUPPORTING TABLE S4 for

Simulation of force spectroscopy experiments on galacturonic acid oligomers

Justyna Cybulska, Agnieszka Brzyska, Artur Zdunek, and Krzysztof Woliński

| **Table S4**. The characterization of six-membered ring conformation in the saccharide units of considered molecules. | | | | | | | | | |
| --- | --- | --- | --- | --- | --- | --- | --- | --- | --- |
| **Oligomer** | **Unit** | **Torsion angle representation (Breces *et al.* 2001)** | | |  | **Spehrical polar coordinate representation** | | | **Conformer** |
|  |  | τ_1_[°] | τ_2_[°] | τ_3_[°] |  | d | θ[°] | φ [°] |  |
| monomer | O4*^a^*O1*^a^* | 49.27 | -50.91 | 55.36 |  | 0.90 | 279 | 5 | ^1^C_4_ |
|  |  |  |  |  |  |  |  |  |  |
| dimer | O4^a^O1^g^ | 53.48 | -48.96 | 49.22 |  | 0.93 | 340 | 7 | ^1^C_4_ |
|  | O1*^g^*O1*^a^* | -67.23 | 33.28 | 28.47 |  | 1.09 | 270 | 92 | ^2^S_O_ |
|  |  |  |  |  |  |  |  |  |  |
| trimer | O4^a^O1*^g^* | 53.53 | -48.58 | 48.99 |  | 0.93 | 341 | 8 | ^1^C_4_ |
|  | O1*^g^*O2*^g^* | -65.67 | 30.68 | 30.68 |  | 1.09 | 269 | 91 | ^2^S_O_ |
|  | O2*^g^*O1*^a^* | -38.44 | 68.18 | -32.77 |  | 1.08 | 327 | 93 | ^1^S_3_ |
|  |  |  |  |  |  |  |  |  |  |
| tetramer | O4^a^O1*^g^* | 49.92 | -44.99 | 47.46 |  | 0.93 | 334 | 11 | ^1^C_4_ |
|  | O1*^g^*O2*^g^* | -30.25 | -26.713 | 61.35 |  | 1.01 | 212 | 90 | ^1^S_5_ |
|  | O2*^g^*O3*^g^* | -24.85 | -31.59 | 61.73 |  | 1.00 | 207 | 90 | ^1^S_5_ |
|  | O3*^g^*O1*^a^* | -63.18 | 39.91 | 18.28 |  | 1.03 | 279 | 91 | ^2^S_O_ |
|  |  |  |  |  |  |  |  |  |  |
| pentamer | O4^a^O1*^g^* | 49.39 | -44.76 | 47.46 |  | 0.93 | 323 | 12 | ^1^C_4_ |
|  | O1*^g^*O2*^g^* | -29.48 | -27.75 | 62.05 |  | 1.01 | 212 | 89 | ^1^S_5_ |
|  | O2*^g^*O3*^g^* | -20.94 | -34.50 | 63.11 |  | 0.99 | 206 | 87 | ^1^S_5_ |
|  | O3*^g^*O4*^g^* | -25.69 | -30.22 | 61.24 |  | 0.99 | 209 | 89 | ^1^S_5_ |
|  | O4*^g^*O1*^a^* | -65.39 | 35.44 | 22.64 |  | 1.02 | 272 | 94 | ^2^S_O_ |
|  |  |  |  |  |  |  |  |  |  |
| hexamer | O4^a^O1*^g^* | 53.9 | -49.87 | 49.66 |  | 0.93 | 342 | 6 | ^1^C_4_ |
|  | O1*^g^*O2*^g^* | -63.3 | 46.27 | 14.73 |  | 1.07 | 286 | 89 | ^2^S_O_ |
|  | O2*^g^*O3*^g^* | -21.91 | -35.15 | 64.67 |  | 1.08 | 334 | 84 | ^5^S_1_ |
|  | O3*^g^*O4*^g^* | -20.58 | -34.74 | 62.82 |  | 0.98 | 205 | 87 | ^5^S_1_ |
|  | O4*^g^*O5*^g^* | -25.24 | -30.65 | 61.29 |  | 0.99 | 208 | 89 | ^5^S_1_ |
|  | O5^g^O1*^a^* | -65.43 | 35.47 | 22.67 |  | 1.03 | 272 | 94 | ^2^S_O_ |
